# Delta radiomic patterns on serial bi-parametric MRI are associated with pathologic upgrading in prostate cancer patients on active surveillance: preliminary findings

**DOI:** 10.3389/fonc.2023.1166047

**Published:** 2023-09-05

**Authors:** Abhishek Midya, Amogh Hiremath, Jacob Huber, Vidya Sankar Viswanathan, Danly Omil-Lima, Amr Mahran, Leonardo K. Bittencourt, Sree Harsha Tirumani, Lee Ponsky, Rakesh Shiradkar, Anant Madabhushi

**Affiliations:** ^1^ Department of Biomedical Engineering, Emory University, Atlanta, GA, United States; ^2^ Picture Health, Cleveland, OH, United States; ^3^ Department of Biomedical Engineering, Case Western Reserve University, Cleveland, OH, United States; ^4^ Fox Chase Cancer Center, Philadelphia, PA, United States; ^5^ Department of Urology, Assiut University, Asyut, Egypt; ^6^ Department of Radiology, University Hospitals, Cleveland Medical Center, Cleveland, OH, United States; ^7^ Department of Urology, University Hospitals, Cleveland Medical Center, Cleveland, OH, United States; ^8^ Atlanta Veterans Administration Medical Center, Atlanta, GA, United States

**Keywords:** active surveillance, prostate cancer, radiomics, magnetic resonance imagining, pathologic upgrade

## Abstract

**Objective:**

The aim of this study was to quantify radiomic changes in prostate cancer (PCa) progression on serial MRI among patients on active surveillance (AS) and evaluate their association with pathologic progression on biopsy.

**Methods:**

This retrospective study comprised *N* = 121 biopsy-proven PCa patients on AS at a single institution, of whom *N* = 50 at baseline conformed to the inclusion criteria. ISUP Gleason Grade Groups (GGG) were obtained from 12-core TRUS-guided systematic biopsies at baseline and follow-up. A biopsy upgrade (AS+) was defined as an increase in GGG (or in number of positive cores) and no upgrade (AS−) was defined when GGG remained the same during a median period of 18 months. Of *N* = 50 patients at baseline, *N* = 30 had MRI scans available at follow-up (median interval = 18 months) and were included for delta radiomic analysis. A total of 252 radiomic features were extracted from the PCa region of interest identified by board-certified radiologists on 3T bi-parametric MRI [T2-weighted (T2W) and apparent diffusion coefficient (ADC)]. Delta radiomic features were computed as the difference of radiomic feature between baseline and follow-up scans. The association of AS+ with age, prostate-specific antigen (PSA), Prostate Imaging Reporting and Data System (PIRADS v2.1) score, and tumor size was evaluated at baseline and follow-up. Various prediction models were built using random forest (RF) classifier within a threefold cross-validation framework leveraging baseline radiomics (*C_br_*), baseline radiomics + baseline clinical (*C_brbcl_*), delta radiomics (*C_Δr_*), delta radiomics + baseline clinical (*C_Δrbcl_*), and delta radiomics + delta clinical (*C_ΔrΔcl_*).

**Results:**

An AUC of 0.64 ± 0.09 was obtained for *C_br_*, which increased to 0.70 ± 0.18 with the integration of clinical variables (*C_brbcl_*). *C_Δr_* yielded an AUC of 0.74 ± 0.15. Integrating delta radiomics with baseline clinical variables yielded an AUC of 0.77 ± 0.23. *C_ΔrΔcl_* resulted in the best AUC of 0.84 ± 0.20 (*p* < 0.05) among all combinations.

**Conclusion:**

Our preliminary findings suggest that delta radiomics were more strongly associated with upgrade events compared to PIRADS and other clinical variables. Delta radiomics on serial MRI in combination with changes in clinical variables (PSA and tumor volume) between baseline and follow-up showed the strongest association with biopsy upgrade in PCa patients on AS. Further independent multi-site validation of these preliminary findings is warranted.

## 1 Introduction

Prostate cancer (PCa) is the second leading cause of cancer-related mortality among men in the United States with nearly 34,500 expected deaths in 2022 (1). However, patients with low or favorable risk PCa do not need radical therapies and can safely be monitored via active surveillance (AS) as demonstrated in the PRIAS study (2), which showed excellent 10-year outcomes in men on AS (2). Patients on AS are usually followed up with serum prostate-specific antigen (PSA) every 6 months, a digital rectal exam (DRE) once a year, and repeat prostate biopsies (PBx) with or without MRI every 12 months or as needed, as per the National Comprehensive Cancer Network (NCCN) guidelines (3–5). The recent guidelines (5) have reestablished AS to be the preferred management strategy for low-risk PCa patients to curb overdiagnosis and overtreatment in men with PCa.

Current standard of care for men on AS is heavily reliant on monitoring via repeat fusion biopsies, which are invasive and expensive; cause discomfort to patients; carry the risk of bleeding, infection, and urinary retention; and are subject to sampling error (6). While MRI-targeted biopsies (7) have been shown to improve PCa localization, there is considerable debate as to whether patients could safely be monitored non-invasively via MRI without the need for invasive biopsies. Anxiety of untreated cancer arising out of this uncertainty (8, 9) is one of the important factors responsible for men discontinuing AS and opt for definitive treatment (2, 10). Besides invasive biopsies, DRE is routinely performed as part of AS (11), which is a source of discomfort and anxiety to patients and associated with false-positive readings (12). Consequently, an accurate, non-invasive imaging-based strategy for identifying candidates for AS and their monitoring is highly desirable.

Studies (13, 14) have shown MRI to be promising in identifying PCa patients suitable for AS; however, this is limited to initial risk stratification. Monitoring of PCa patients on AS using serial MRI showed a high negative predictive value but a moderate positive predictive value and currently cannot replace biopsies. Radiologist assigned Prostate Imaging Reporting and Data System (PIRADS v2.1) scores have been studied (15) for patient triage to minimize repeat biopsies and improve quality of life; however, no objective guidelines exist for non-invasive monitoring. The Prostate Cancer Radiological Estimation of Change in Sequential Evaluation (PRECISE) (16) criteria have been proposed to monitor PCa progression on MRI using standardized guidelines. While upgrading in MRI-negative men was substantially lower compared to MRI-positive men, PRECISE criteria alone were insufficient to monitor and predict upgrading in MRI-positive men (13). Confirmatory systematic and targeted biopsies are still required to monitor patients on AS, despite the use of serial MRI. Clinical factors including PSA density have been studied and demonstrated to identify candidates for AS (17). PSA density in conjunction with MRI has been explored as a dynamic risk prediction strategy to monitor patients on AS (18). However, conclusive and objective guidelines for risk estimation of disease progression on AS are limited. Therefore, there is currently a critical need to improve MRI-based interpretation of PCa progression in conjunction with routine clinical factors including PSA to achieve non-invasive monitoring for AS.

Radiomics from prostate MRI have shown significant promise in characterization and risk stratification of PCa (19). Radiomics involves high-throughput extraction of quantitative measurements of subtle image texture and heterogeneity patterns using advanced image processing techniques that are not apparent on routine visual inspection. Bi-parametric MRI (bpMRI) that includes T2W and DWI sequences was shown (20, 21) to be efficient and non-inferior in diagnostic performance in comparison to multiparametric MRI (which includes contrast enhanced MRI). We hypothesize that “delta” changes in the progression of PCa on AS can be quantified using radiomic features on serial bpMRI. In this preliminary proof-of-concept study, we explore radiomic features from PCa regions on serial bpMRI (baseline and follow-up) along with routine clinical parameters for their association with PCa progression on AS. We employ a single institutional dataset consisting of *N* = 50 PCa patients on AS who underwent baseline followed by repeat 3T MRI on *N* = 30 patients along with confirmatory systematic biopsies. We compare our integrated approach combining radiomics with clinical variables against radiologist assigned PIRADS v2.1 scores in predicting biopsy upgrade for PCa AS.

## 2 Methods

### 2.1 Patient selection

**Figure 1:**
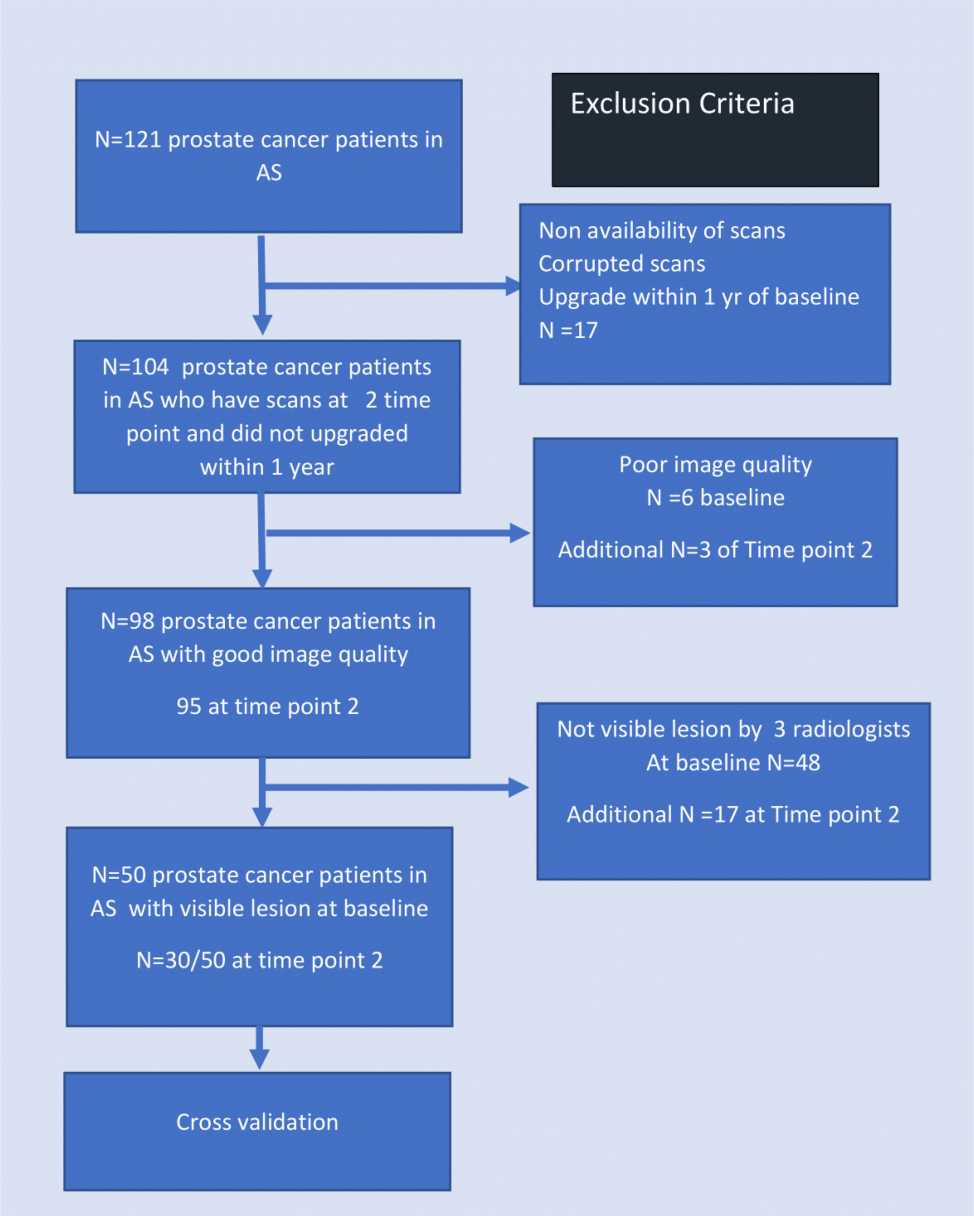
Summary of patient selection from an initial cohort of 121 patients.

This HIPAA-compliant, retrospective study was approved by the local institutional review board (IRB) that waived the need for informed consent. A chart review was performed to include patients diagnosed with biopsy-proven PCa between 2012 and 2020 according to the following criteria: (a) patients with histopathologically documented PCa who were enrolled on AS, (b) availability of 3T MRI and systematic biopsy at baseline, (c) followed up with PSA measurements and biopsies at least every 12 months for ≥3 years, and (d) including at least one additional 3T MRI between 18 and 36 months (see **Figure 1**). Patients with (a) imaging artifacts, (b) non-visible lesions, (c) negative biopsy at baseline, (d) non-availability of at least T2W and DWI sequences at baseline and follow-up, (e) discordant imaging and pathology findings, (f) different location of positive biopsy core at baseline and follow-up, and (g) disappeared lesion/newly detected location at follow-up were excluded from the study. All images were acquired with 3T MRI scanners (Siemens Skyra and Philips Ingenia) using pelvic phased array surface coil. The acronyms used in this manuscript are provided in **Table 1**. Detailed imaging parameters and characteristics are provided in **Table 2**. The International Society of Urological Pathology (ISUP) Gleason Grade Groups (GGG) were determined from 12-core transrectal ultrasound (TRUS)-guided systematic biopsies at baseline and follow-up. A biopsy upgrade (AS+) was defined as an increase in GGG from baseline 1 to ≥2 (increase in number of positive cores for baseline GGG = 2) at follow-up and no upgrade (AS−) when GGG remained the same. Detailed information about the patient cohort is provided in **Table 3**.

**Table 1:** List of acronyms used throughout the manuscript.

Name	Abbreviation
**Baseline Radiomic Features**	*F_r_*
**Baseline Clinical Features**	*F_cl_*
**Delta Radiomic Features**	*F_Δr_*
**Baseline Imaging Model**	*C_br_*
**Baseline Clinical Model**	*C_bcl_ x €[,PSA, Tumor Volume]_*
**Baseline Integrated Model**	*C_brbcl_*
**Delta Radiomic Model**	*C_Δr_*
**Integrated Delta Radiomic and Baseline Model**	*C_Δrbcl_*
**Delta Integrated Model**	*C_ΔrΔcl_*
**Prostate Imaging Reporting & Data System**	PIRADS
**Prostate Biopsy**	PBx
**Prostate Cancer**	PCa
**Clinically Significant Prostate Cancer**	csPCa
**Active Surveillance**	AS

**Table 2:** MRI parameters.

Parameters	Scanner
**Manufacturer**	(Siemens Healthcare, Erlangen, Germany), (Philips Medical Systems, Best, Netherlands)
**Model**	(3T Skyra), 3T Ingenia
*T2-weighted MR imaging*	
**Repetition time/echo time (TR/TE)**	3,894–8,740/90–150
** Reconstruction spatial resolution (mm^3^)**	0.56–1.25 × 0.56–1.25 × 2–3.5
*Diffusion-weighted imaging*	
**Repetition time/echo time (TR/TE)**	3,656–11,300/64–83
**Reconstruction spatial resolution (mm^3^)**	0.87–1.5 × 0.87–1.5 × 3–3.5
***b*-values (s/mm^2^)**	0, 500, 1,000, 1,500, 2,000–0, 400, 900, 1,500

**Table 3:** Patient demographics.

Clinical Predictor	AS+ (n = 28)	AS− (n = 22)	p-value
	Median value [range]		
**Age (years)**	68 [52–80]	66 [52–77]	
**PSA at diagnosis (mg/ml)**	5.91 [2.36–16.98]	4.61 [13.61–1.11]	<0.0005
**PIRADS at diagnosis**	[3–4]	[3–5]	<0.0005
**PSA at follow-up**	8.165 [2.35–18.93]	6.46 [4–11.2]	
**PIRADS at follow-up**	[3–5]	[3–5]	
**Time interval between diagnosis and upgradation (months)**	17.5 [8–45]	15.5 [4–57]	

### 2.2 Prostate cancer delineations on MRI

Two board-certified genitourinary radiologists with greater than 10 years of experience in prostate imaging reviewed all the MRIs and delineated PCa regions of interest (ROI) on T2W and ADC. The readers were provided with the baseline and follow-up scans separately and were blinded to the relationship between the two sets of scans. They were also blinded to pathology results as well as positive core locations to avoid radiologists’ bias in identifying PCa lesions. The readers were provided with high-resolution T2W, high *b*-value DWI and ADC images in the axial plane along with DCE sequences when available. ROI delineations were made by the reader on the T2W slice using the MD.AI software (22). The readers could choose to delineate a lesion if visible or label the scan as negative on MRI or of poor diagnostic quality. The readers assigned PIRADS v2.1 scores to all lesions. A subset of patients was assigned to both radiologists to evaluate inter-reader variations in PCa delineation as well as PIRADS v2.1 scoring.

ROIs delineated by readers were later matched against positive biopsy core location obtained from systematic biopsies. Patients whose MRI scans were not assigned a PIRADS v2.1 score due to poor image quality or those that did not match with positive biopsy core locations were excluded from the study. In patients with multiple lesions, the lesion corresponding to the highest PIRADS v2.1 score and corresponding positive biopsy core location was included. The above-described rigorous steps were followed to ensure that no reader bias was introduced while identifying PCa ROIs on MRI.

### 2.3 Preprocessing of MRI

Prostate cancer ROIs were delineated by the radiologists on T2W as they provide the greatest anatomic resolution. ADC was co-registered to T2W using a rigid transformation using the Elastix toolbox (23, 24) (specific parameters are provided in the **Supplementary Material**), which was used to map PCa ROIs onto ADC. These ROIs were verified by radiologists and modified if they were not correctly aligned with the corresponding suspicious region on ADC. When there was significant deformation in ADC that could not be accounted through rigid transformation, separate ROIs were delineated for ADC.

Intra-patient intensity drift artifacts may cause image intensities to lack in tissue-specific meaning. This was corrected using a previously presented MRI intensity standardization method (25), which normalizes the histogram of intensities from a given region against a template image delivering tissue-specific intensity range. A pre-identified template using a subset of scan was used as a reference against which all scans in this study were mapped. This ensures that T2W measurements were normalized and reflect a tissue-specific meaning. Although we standardize the T2 scans, we did not standardize ADC scans since they are from single site and quantitative map.

### 2.4 Radiomic feature extraction

From the radiologist-delineated PCa ROIs on T2W and ADC, a set of 252 radiomic features were extracted using in-house software. These include first- and second-order statistics (mean, median, standard deviation, and range); 13 Haralick features (26), which are statistics derived from gray-level co-occurrence matrix (entropy, energy, inertia, inverse difference moment, marginal distributions, correlation, information measures of correlation, sum of average, sum of variance, sum of entropy, difference of average, difference of variance, difference of entropy, and contrast) computed at a window size of 3 × 3, 5 × 5, and 7 × 7; 5 Gabor features; and 1 CoLIAGe (27) feature. Radiomic features were extracted and processed in agreement with the image biomarker standardization initiative (IBSI) criteria (28). Features were extracted on each 2D slice of T2W and ADC volumes with a delineated PCa ROI. Distribution statistics (mean, variance, skewness, and kurtosis) of each of these features over the entire lesion were computed to obtain a single feature vector per lesion.

Radiomic features were extracted from the baseline as well as follow-up bpMRI. Delta radiomics (*F_Δr_*) were computed as the difference in feature vector between baseline (*F_br_*) and follow-up, which quantify radiomic changes in lesion heterogeneity. Additional details regarding these features are provided in the supplementary section.

### 2.5 Radiomic feature selection and classification

**Figure 2:**
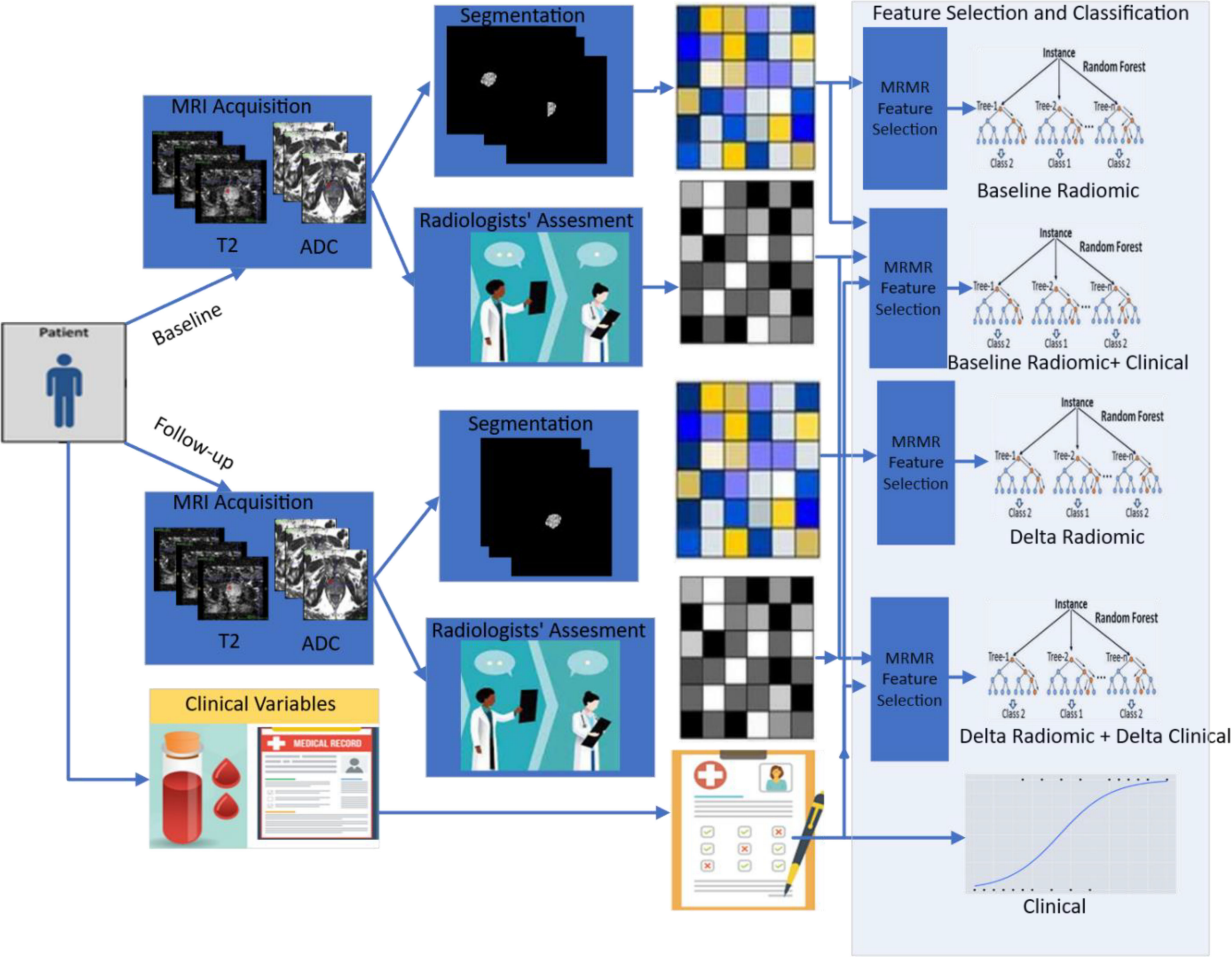
Pipeline used in this study illustrating radiomics from serial prostate biparametric MRI to predict pathologic progression in prostate cancer patients on AS. Radiomic features are extracted from prostate cancer regions of interest delineated by experienced radiologists at baseline and follow-up, which are then integrated with clinical variables in different combinations to build machine learning models (baseline radiomics, baseline radiomics + clinical, delta radiomics, delta radiomics + delta clinical, and clinical).

Radiomic features that were correlated with each other were discarded using Pearson’s correlation coefficient at a threshold of 0.90. The best set of discriminating features between AS+ and AS− patients were identified using the maximum relevance and minimum redundancy (mRMR) feature selection method (29), which has previously shown to be useful in identifying the optimal set of radiomic features for PCa risk stratification. A random forest (RF) machine learning classifier was used to train radiomic features in conjunction with mRMR feature selection to predict biopsy upgrade in AS patients. Clinical variables including PSA, tumor volume, and age were collected from electronic health records, and their association with biopsy upgrade was evaluated. Wilcoxon rank-sum test was employed to determine statistically significant (*p* < 0.05) features between AS+ and AS− cohorts. Different machine learning classifiers were constructed using radiomic features—(a) baseline radiomics (*C_br_*), (b) delta radiomics (*C_Δr_*), (c) integrated baseline radiomics and clinical variables (*C_brbcl_*), (d) integrated delta radiomics and baseline clinical (*C_Δrbcl_*), and (e) integrated delta radiomics and delta clinical (*C_ΔrΔcl_*). We also compared the performance of our best machine learning classifier with a radiologist-assigned PIRADS v2.1 score at baseline and follow-up. A schematic diagram illustrating the methodology is shown in **Figure 2**.

### 2.6 Statistical analysis

Each classification model (*C_br_*, *C_bcl_*, *C_Δr_*, *C_Δrbcl_*, *C_Δrbcl_*, and *C_ΔrΔcl_*) was trained using 100 runs of threefold cross-validation. Because of the small sample size, we did not employ an independent hold out set in our study. The classification performance was evaluated in terms of area under the receiver operating characteristics curve (AUC), sensitivity, specificity, positive predictive value (PPV), and negative predictive value (NPV). The performance of developed models was also compared with clinical attributes (PSA, tumor volume, and age) and radiologists’ assessment (PIRADS v2.1) using univariate and multivariate logistic regression. Computational analysis was performed in MATLAB v2021b (Nattick, MA, USA) and R v4.1.3.

## 3 Results

Based on the inclusion criteria, *N* = 121 PCa patients were identified with mpMRI scans available at baseline and follow-up. Of these, *N* = 17 patients were excluded due to either poor-quality MRI, non-availability of scans, or upgraded within 1 year after baseline. Among the remaining ones, radiologists did not find any visible lesion in *N* = 48 patient studies and were excluded. Another *N* = 6 were discarded due to poor image quality following PIRADS v2.1 guidelines. Of the *N* = 50 selected patients, *N* = 28 experienced biopsy upgrade (AS+) while others retained biopsy GGG (AS−). Of these, *N* = 30 patients had a follow-up MRI of adequate quality available, of which *N* = 19 experienced pathologic upgrading (AS+).

### 3.1 Radiomics from baseline prostate MRI for predicting AS+

**Figure 3:**
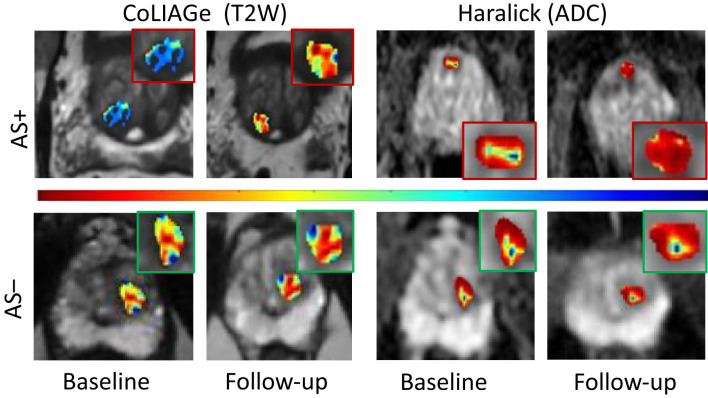
Radiomic feature maps of prostate cancer at baseline MRI and follow- up MRI belonging to patients with pathologic progression (AS+) and those without (AS−). CoLIAGe features from T2W MRI (columns 1 and 2) and Haralick’s feature from ADC maps (columns 3 and 4) illustrated differential heterogeneity in PCa appearance for AS+ and AS− patients between baseline and follow-up. The inset shows radiomic heat maps of the prostate cancer lesion in greater detail. Hotter colors (red) indicate higher heterogeneity associated with radiomic features and cooler colors (blue) indicate lower heterogeneity. Patients experiencing AS+ show a significant increase in radiomic feature expression between follow-up and baseline compared to patients with no upgrade (AS−).

In this experiment, we aimed to evaluate radiomics of PCa lesions at baseline bpMRI in predicting pathologic upgrade in AS patients (AS+). Radiomic features at baseline (*F_br_*) including Haralick (sum of entropy, sum of variance, and difference entropy) features from T2W and Haralick and CoLlAGe energy features from ADC maps were significantly associated with AS+ (*p* < 0.05) (**Table 4**). We observed that gray-level and gradient-level co-occurrence (quantified by Haralick and CoLlAGe features) from baseline bpMRI (T2W and ADC) were associated with pathologic progression on AS as illustrated in terms of radiomic feature maps in **Figure 3**. These features essentially capture intensity and gradient-based heterogeneity through statistics of corresponding co-occurrence matrices. Radiomic classifier (*C_br_*) trained using baseline radiomics (*F_br_*) from ADC and T2W resulted in AUC = 0.56 ± 0.05 and 0.58 ± 0.06, respectively. Combined radiomic features from T2W and ADC resulted in improved classification performance AUC = 0.64 ± 0.09. The improvement is statistically significant (*p* < 0.05).

**Table 4:** Top three frequently selected features with their *p*-value.

Experiment	Imaging type	Feature name	p-value
***C_br_***	T2W	Kurtosis of Haralick’s sum of entropy	0.0067
		Kurtosis of Haralick’s sum of variance	0.0158
		Mean of Haralick’s difference entropy	0.0028
	ADC	Skewness of Haralick’s energy	0.1755
		Mean of CoLiAGe	0.0325
		Variance of Haralick’s energy	0.0325
***C_Δr_***	T2W	Mean of Haralick’s difference entropy	0.0032
		Kurtosis of Haralick’s sum of variance	0.0062
		Skewness of Haralick’s difference entropy	0.0007
	ADC	Mean of Haralick’s entropy	0.0050
		Kurtosis of Haralick’s energy	0.0617
		Kurtosis of diagonal difference	0.0302

### 3.2 Delta radiomics from prostate MRI for prediction of AS+

In this experiment, we evaluated the association between delta radiomics *F_Δr_* (computed as the difference in radiomics of PCa lesion between baseline and follow-up MRI) and pathologic upgrading on biopsy. Delta radiomics *F_Δr_* including Haralick features (difference of entropy and sum of variance) from T2W and Haralick features (entropy, energy, and diagonal difference) from ADC were observed to be associated with AS+. Machine learning classifier (*C_Δr_*) trained using delta radiomic features from T2W and ADC resulted in AUCs of 0.71 ± 0.08 and 0.72 ± 0.12, respectively. Combining T2W and ADC delta radiomics resulted in an AUC = 0.74 ± 0.15. The classification performance metrics are provided in **Table 5**.

**Table 5:** Performance evaluation of predictive power of imaging, clinical model from baseline, as well delta radiomics in terms of accuracy, sensitivity, specificity, PPV (positive predictive value), NPV (negative predictive value) maximizing sensitivity, and specificity.

	Experiments	Accuracy	Sensitivity	Specificity	PPV	NPV
**Baseline** **(*n* = 50)**	T2W	0.50 ± 0.09	0.65 ± 0.21	0.41 ± 0.13	0.43 ± 0.05	0.71 ± 0.06
	ADC	0.56 ± 0.10	0.66 ± 0.22	0.50 ± 0.29	0.50 ± 0.15	0.77 ± 0.11
	T2W + ADC (*C_br_*)	0.57 ± 0.10	0.61 ± 0.27	0.53 ± 0.28	0.50 ± 0.14	0.73 ± 0.11
	PSA	0.50 ± .010	0.67 ± 0.31	0.40 ± 0.33	0.42 ± 0.06	0.75 ± 0.13
	PIRADS	0.71 ± .004	0.37 ± 0.08	0.91 ± 0.09	0.75 ± 0.13	0.71 ± .002
	Tumor volume	0.79 ± 0.03	0.69 ± 0.04	0.89 ± 0.05	0.84 ± 0.05	0.77 ± 0.02
	Imaging + Cl (*C_brbcl_*)	0.59 ± 0.08	0.71 ± 0.21	0.51 ± 0.24	0.49 ± 0.11	0.80 ± 0.11
**Delta Radiomics** **(*n* = 30)**	T2W	0.72 ± 0.07	0.81 ± 0.19	0.67 ± 0.16	0.62 ± 0.11	0.88 ± 0.09
	ADC	0.73 ± .07	0.82 ± 0.15	0.68 ± 0.15	0.62 ± 0.09	**0.89 ± 0.07**
	T2W + ADC (*C_Δr_*)	0.75 ± 0.06	0.78 ± 0.17	0.73 ± 0.13	0.65 ± 0.11	0.87 ± 0.088
	Change in PIRADS	0.50 ± 0.05	0.67 ± 0.04	0.45 ± 0.03	0.58 ± 0.02	0.58 ± 0.01
	Imaging + Cl at baseline (*C_Δrbcl_*)	0.67 ± 0.08	0.76 ± 0.15	0.60 ± 0.20	0.64 ± 0.10	0.78 ± 0.11
	Imaging+ delta Cl (*C_ΔrΔcl_*)	**0.81 ± 0.05**	**0.83 ± 0.13**	**0.78 ± 0.16**	**0.77 ± 0.13**	0.87 ± 0.08

C_br_ = Baseline radiomics model, C_brbcl_ = Baseline radiomics + baseline clinical model, C_Δr_ = Delta radiomics model, C_Δrbcl_ = Delta radiomics + baseline clinical, C_ΔrΔcl_ = Delta radiomics + delta clinical model. The bold face denotes the best performance for each performance evaluation index.

### 3.3 Integrated nomogram with clinical variables, baseline, and delta radiomics

We integrated clinical parameters including PSA, tumor volume, and age with the risk scores estimated from classifiers (*C_br_*, *C_Δr_*, and *C_bcl_*) to develop integrated classifiers *C_Δrbcl_*, *C_Δrbcl_*, and *C_ΔrΔcl_* for predicting AS+. Clinical parameters including PSA, tumor volume, age at diagnosis, and PIRADS v2.1 at baseline were evaluated for their association with AS+. On univariate analysis, PIRADS v2.1, PSA, and tumor volume were found to be significantly associated (*p* < 0.05) with AS+, resulting in AUCs of 0.62 ± 0.1, 0.61 ± 0.08, and 0.67 ± 0.12, respectively. An integrated model (*C_brbcl_*) combining baseline radiomics and significant baseline clinical parameters (PSA and tumor volume) delivered an AUC of 0.70 ± 0.18. In comparison, PIRADS v2.1 scores from baseline resulted in an AUC = 0.62 ± 0.05, indicating that our integrated model *C_brbcl_* significantly outperformed PIRADS v2.1 scores.

**Figure 4:**
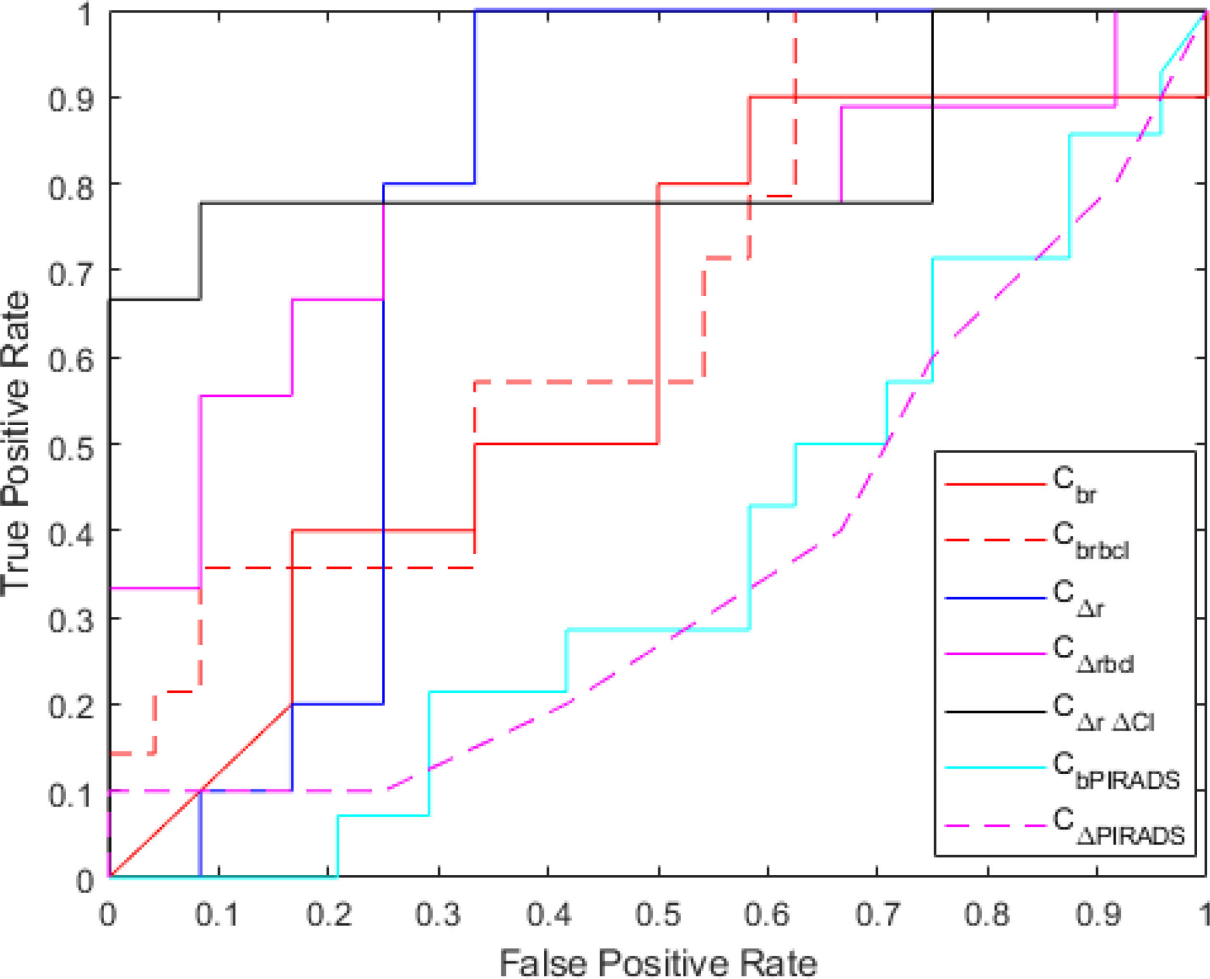
Receiver operating characteristics curve for baseline radiomics (*C_br_*), baseline radiomics + baseline clinical (*C_brbcl_*), delta radiomics (*C_Δr_*), delta radiomics + baseline clinical (*C_Δrbcl_*), delta radiomics + delta clinical (*C_Δr Δcl_*), baseline PIRADS (*C_bPIRADS_*), and delta PIRADS (*C_ΔPIRADS_*).

We integrated significant clinical variables (PSA and tumor volume) at baseline with *F_Δr_* to build an integrated model (*C_Δrbcl_*) that resulted in an AUC of 0.77 ± 0.23. Finally, we developed another integrated model (*C_ΔrΔcl_*) combining delta radiomics and delta clinical variables (PSA and tumor volume) delivering the highest AUC of 0.84 ± 0.20. *C_ΔrΔcl_* also significantly outperformed PIRADS v2.1 scores at baseline and follow-up in predicting AS+ (AUC = 0.62 and 0.67, respectively, for baseline and follow-up). ROC curves resulting from *C_br_*, *C_Δr_*, *C_brbcl_ C_Δrbcl_*, and *C_ΔrΔcl_* are illustrated in **Figure 4**. Detailed results corresponding to these classifiers are provided in **Table 5**. The improvement obtained by combining delta radiomics and delta clinical was statistically significant over other combinations. Additionally, specificity, PPV, and NPV metrics computed at 90% sensitivity are provided in **Table 6**.

**Table 6:** Specificity, PPV, and NPV of different predictive models at 90% sensitivity.

	Experiments	Specificity	PPV	NPV
**Baseline** **(*n* = 50)**	T2W	0.21	0.39	0.71
	ADC	0.38	0.44	0.82
	T2W + ADC (*C_br_*)	0.41	0.53	0.83
	PSA	0.08	0.35	0.50
	PIRADS	0.17	0.33	0.50
	Tumor volume	0.08	0.35	0.50
	Imaging + Cl (*C_brbcl_*)	0.17	0.39	0.80
**Delta Radiomics** **(*n* = 30)**	T2W	0.17	0.44	0.67
	ADC	0.08	0.42	0.50
	T2W + ADC (*C_Δr_*)	0.25	0.47	0.75
	Imaging + Cl at baseline (*C_Δrbcl_*)	0.42	0.53	0.83
	Imaging + delta Cl (*C_ΔrΔcl_*)	0.75	0.73	0.90

## 4 Discussion

The current standard of care for identifying and monitoring PCa patients on AS involves PSA tests, invasive biopsies, and DRE (3–5). While serial MRI is being widely incorporated into the AS setting, several long-term studies (10, 30) have indicated that serial MRI alone cannot be reliably used to monitor progression. This is due to its low positive predictive value and presence of invisible and benign confounding tumor appearances (10, 30). In this preliminary proof-of-concept study, we explored the role of radiomics from baseline and follow-up serial bi-parametric prostate MRI in conjunction with clinical parameters in order to evaluate association with pathologic progression on biopsy. A biopsy upgrade (AS+) was defined as an increase in GGG from baseline 1 to ≥2 (increase in number of positive cores for baseline GGG = 2) at follow-up and no upgrade (AS−) when GGG remained the same. To the best of our knowledge, our study is one of the first attempts at exploring the combination of change in routinely acquired clinical parameters with handcrafted radiomics from serial MRI to predict pathologic progression in AS patients. We observed that radiologic progression of PCa captured by delta radiomic features (specifically Haralick and CoLlAGe features) between baseline and follow-up MRI was significantly associated with pathologic progression on biopsy (AS+). These features essentially capture intensity and gradient-based heterogeneity through statistics of corresponding co-occurrence matrices. A machine learning classifier trained using delta radiomic features showed stronger association with biopsy upgrade compared to baseline radiomics (AUC = 0.74 vs. 0.71) and radiologist-assigned PIRADS v2.1 scores (AUC = 0.62). Integrating delta radiomics with changes in clinical parameters (PSA and tumor volume) between baseline and follow-up resulted in the best classification model in predicting AS+ (AUC = 0.81).

Baseline bp-MRI has been shown to be promising in predicting pathologic upgrading in patients on AS (14). Studies (31, 32) have shown that PIRADS ≥3 have a positive predictive value of 35%–40% for reclassification at 3 years. While this is insufficient to recommend the use of MRI alone for AS (10, 33), it suggests that features associated with aggressive PCa that would go on to experience biopsy progression can be observed on MRI. In addition, the presence of MRI invisible PCa lesions precludes the possibility of obviating confirmatory biopsies (32) as indicated by the MRIAS trial (32). Since patients on AS are characterized by low-risk disease, radiomic features capable of capturing subtle, subvisual characteristics of future progression need to be identified. We observed that quantitative radiomic features related to intensity and gradient-based heterogeneity on MRI were associated with pathologic upgrading in PCa patients on AS. Interestingly, in addition to the baseline radiomics, we observed that PIRADS v2.1, PSA, and tumor volume at baseline were strongly associated with AS+ (AUC = 0.61, 0.62, and 0.67). However, sensitivity of PIRADS v2.1 was found to be poor in our study, suggesting that PIRADS v2.1 may miss detecting pathologic progression (AS+). Although PIRADS v2.1 was not developed for predicting pathologic upgrade for AS, PIRADS ≥3 at follow-up and increase in PIRADS score of a lesion between baseline and follow-up are a non-invasive estimation of clinically significant PCa at follow-up. This indicates that radiological progression may be associated with increase in biopsy GGG on AS.

A few recent studies have explored artificial intelligence (AI)-based approaches using baseline MRI for predicting biopsy upgrading in PCa patients on AS. Sushentsev et al. (31) demonstrated that radiomics from baseline bpMRI were significantly associated with pathologic progression in PCa patients. However, they noticed incremental benefit with the addition of clinical parameters. Another study by Xie et al. investigated the role of radiomics from ADC maps to predict upgrading in Gleason score from TRUS-guided biopsies to radical prostatectomy (34). While Xie et al.’s study (34) did not directly address progression on repeat biopsy, they demonstrated that radiomic features from screening MRI can differentiate clinically significant and insignificant PCa.

Serial MRI is being actively explored for monitoring tumor progression in patients on AS as opposed to protocol-based biopsies (35). However, lesion visibility and limited PIRADS v2.1 accuracy continue to limit its potential for non-invasive monitoring. In our study, we observed that radiological tumor progression quantified using delta radiomics (intensity co-occurrence features from T2W and ADC) showed significant association with pathologic progression (**Figure 3**). These features reflect intensity and gradient-based subvisual heterogeneity attributes that are potentially associated with pathologic progression. We demonstrated that the delta-radiomics risk score is associated with clinical trajectory of the cancer lesion towards pathologic progression on AS and is better associated with biopsy upgrade compared to PIRADS or change in PIRADS on follow-up.

A closely related study by Sushentsev et al. also demonstrated that delta radiomics from PCa ROIs on serial bpMRI were associated with pathologic progression in patients on AS (36). They also observed that Haralick (co-occurrence) features from T2W and ADC were associated with PCa progression. However, in our study, we leveraged novel radiomic features including CoLlAGe, which aims to quantify gradient-based tissue heterogeneity; these features have been previously shown to be associated with aggressive PCa (37–39). While the Sushentsev study (36) compared delta radiomics from MRI against PRECISE score (16) for predicting pathologic progression, we integrated delta radiomics with delta changes in PSA and tumor volume; this combined model yielded the best prediction results. We also evaluated radiomic features at baseline and in combination with routine clinical parameters (PSA and tumor volume) for their association with AS+, corroborating the idea of dynamic monitoring using MRI and PSA dynamics (40). We ensured strict controls and blinded our readers to pathologic findings to minimize reader bias; it is unclear whether such precautions to mitigate potential bias were invoked in the study of Sushentsev et al. (36). AUC reported in their study (36) was comparable to our *C_ΔrΔcl_* model; however, their approach (36) relies on a qualitative PRECISE score that is susceptible to inter-observer variability. This is different compared to quantitative radiomic texture measurements. Moreover, our dataset is different from the dataset used in Sushentsev et al. (36); thus, it may not be possible to perform a precise comparison.

Another related study was the one by Roest et al. who developed a deep learning model using serial MRI for monitoring PCa progression (41). They built a U-Net-based deep learning model to detect clinically significant prostate cancer (csPCa) at baseline and follow-up MRI, and extract differential tumor volume and csPCa likelihood scores, which were then used to train a supervised machine learning model to detect csPCa. They trained the csPCa detection model on patients with screening MRI who were not enrolled on AS and evaluated it for identification of csPCa on AS patients with serial MRI. However, their method relies on deep learning for detection of csPCa. Despite recent works aimed at improving interpretability (42–44), deep learning essentially is a black box-based approach that may not allow for biological interpretation of signatures associated with PCa progression. Moreover, deep learning approaches were found to fail to converge and generalize in the absence of a large dataset as demonstrated in a recent study of machine learning for treatment response prediction in ophthalmology images by Dong et al. (45).

Our approach, on the other hand, involved the use of handcrafted radiomic features explicitly associated with PCa progression from radiologist-identified PCa ROIs on MRI; these features were subsequently found to be associated with pathologic progression. Similar to our study, the findings from Roest et al. indicate that computationally derived features from serial prostate MRI can enable non-invasive surveillance. Serial MRI in conjunction with PSA kinetics is becoming increasingly popular as an alternative to unnecessary repeat biopsies (18). Results from our study also reflect the potential opportunity for dynamic monitoring using repeat MRI and PSA density trends for men with PCa on AS (35).

Our study, however, had several limitations. Firstly, the sample size of the dataset used in the development of baseline and delta radiomic models was small. A large number of patients were excluded due to the presence of MRI non-visible PCa lesions. Owing to the small sample size, we could only report cross-validation results without testing on a hold-out validation set. However, our sample size is comparable to other radiomic studies in the context of AS including those of Sushentsev et al. (31, 36) and Algohary et al. (46). Even the study by Roest et al. (41) employed a comparably sized set of patients. Additionally, our findings suggest the presence of a strong association between delta radiomics in combination with a change in clinical variables and biopsy upgrade. Our results were in agreement with those reported by other groups (31, 34). Second, pathologic progression was estimated using systematic biopsy, which may not be as accurate as MRI-targeted biopsy (47). However, we ensured that the positive biopsy core locations matched with the location of blinded ROI delineations by the radiologists. We also ensured that the same lesion at baseline was being followed up on serial MRI. Thirdly, two readers were involved in this study and inter-observer differences in PIRADS v2.1 and ROI delineations may have impacted radiomic feature extraction. However, a small subset of cases (*N* = 15) were read by the two readers and a reasonably good inter-reader agreement in ROI (kappa = 0.80) was determined. In future work, we will also seek to quantitatively assess the impact of inter-reader variation on a larger number of cases. Fourth, the difference between baseline and follow-up MRI was not consistent across all patients since this was a retrospective cohort. Nevertheless, we limited the follow-up MRI at 3 years with a range of ±12 months to ensure a relatively homogeneous follow-up. Lastly, radiologists missed a significant number of PCa lesions at baseline due to poor diagnostic quality or the presence of non-visible lesions given that they were blinded to biopsy results. This was made to ensure that no reader bias was introduced in obtaining PCa ROIs on MRI.

In summary, preliminary findings from our single-center study suggest that quantitative radiomic features derived from baseline and serial MRI are associated with biopsy upgrade on AS. Delta radiomics from serial MRI in conjunction with routine clinical parameters (including PSA and tumor volume) may be used to non-invasively predict pathologic progression in PCa patients on AS. Our findings align with those from previous studies (36, 41, 48), which suggest that machine learning and deep learning approaches with prostate MRI can enable non-invasive monitoring of patients on AS. Future directions will involve large-scale multisite validation of delta radiomics approaches from serial MRI, automated and reliable pipelines for lesion detection, and prospectively validating these approaches in a clinical setting.

## Data availability statement

The raw data supporting the conclusions of this article will be made available by the authors following institutional guidelines, without undue reservation. Requests to access the data should be directed to the corresponding authors.

## Ethics statement

The studies involving human participants were reviewed and approved by University Hospitals Cleveland Medical Center, Cleveland 06-16-30C. The patients/participants provided their written informed consent to participate in this study.

## Author contributions

AbM contributed to the experimental design, analysis, software, writing. AH and JH contributed to the software, data analysis. VV helped with the data acquisition, data preprocessing, and writing. DL and AnM contributed data collection. LB and ST contributed to the imaging interpretation data preparation, and writing. acquisition, funding, and clinical interpretation. LP contributed to the data collection and conception of idea. AnM and RS contributed to the conception of the idea, experimental design, supervision, writing, and funding. All authors contributed to the article and approved the submitted version.
