# Supplementary material for: Delta radiomic patterns on serial bi-parametric MRI are associated with pathologic upgrading in prostate cancer patients on active surveillance: preliminary findings
Source: Front Oncol. 2023 Sep 5;13:1166047. doi: 10.3389/fonc.2023.1166047 (PMC10508842; doi:10.3389/fonc.2023.1166047)
Supplement: Supplementary file 1 [file DataSheet_1.docx]

|  | Experiments | Specificity | PPV | NPV |
| --- | --- | --- | --- | --- |
| Baseline  (n=50) | T2w | 0.21 | 0.39 | 0.71 |
|  | ADC | 0.38 | 0.44 | 0.82 |
|  | T2w+ADC (*C_br_*) | 0.41 | 0.53 | 0.83 |
|  | PSA | 0.08 | 0.35 | 0.50 |
|  | PIRADS | 0.17 | 0.33 | 0.50 |
|  | Tumor Volume | 0.08 | 0.35 | 0.50 |
|  | Imaging+ Cl (*C_brbcl_*) | 0.17 | 0.39 | 0.80 |
| Delta Radiomics  (n=30) | T2w | 0.17 | 0.44 | 0.67 |
|  | ADC | 0.08 | 0.42 | 0.50 |
|  | T2w+ADC (*C_Δr_*) | 0.25 | 0.47 | 0.75 |
|  | Imaging+Cl at baseline (*C_Δrbcl_*) | 0.42 | 0.53 | 0.83 |
|  | Imaging+ delta Cl (*C_ΔrΔcl_*) | 0.75 | 0.73 | 0.90 |

**Supplemental-I: Performance evaluation at different sensitivity**

Specificity, PPV, and NPV computed at 90% sensitivity

|  | Experiments | Specificity | PPV | NPV |
| --- | --- | --- | --- | --- |
| Baseline  (n=50) | T2w | 0.25 | 0.36 | 0.71 |
|  | ADC | 0.46 | 0.46 | 0.79 |
|  | T2w+ADC (*C_br_*) | 0.59 | 0.59 | 0.73 |
|  | PSA | 0.25 | 0.38 | 0.67 |
|  | PIRADS | 0.42 | 0.39 | 0.67 |
|  | Tumor Volume | 0.21 | 0.37 | 0.63 |
|  | Imaging+ Cl (*C_brbcl_*) | 0.38 | 0.42 | 0.80 |
| Delta Radiomics  (n=30) | T2w | 0.33 | 0.46 | 0.67 |
|  | ADC | 0.17 | 0.38 | 0.40 |
|  | T2w+ADC (*C_Δr_*) | 0.33 | 0.47 | 0.67 |
|  | Imaging+Cl at baseline (*C_Δrbcl_*) | 0.67 | 0.67 | 0.8 |
|  | Imaging+ delta Cl (*C_ΔrΔcl_*) | 0.83 | 0.78 | 0.83 |
|  |  |  |  |  |

Specificity, PPV, and NPV computed at 80% sensitivity

|  | Experiments | Specificity | PPV | NPV |
| --- | --- | --- | --- | --- |
| Baseline  (n=50) | T2w | 0.33 | 0.36 | 0.62 |
|  | ADC | 0.5 | 0.43 | 0.71 |
|  | T2w+ADC (*C_br_*) | 0.75 | 0.67 | 0.75 |
|  | PSA | 0.29 | 0.37 | 0.64 |
|  | PIRADS | 0.54 | 0.42 | 0.68 |
|  | Tumor Volume | 0.25 | 0.36 | 0.60 |
|  | Imaging+ Cl (*C_brbcl_*) | 0.46 | 0.43 | 0.73 |
| Delta Radiomics  (n=30) | T2w | 0.5 | 0.5 | 0.67 |
|  | ADC | 0.25 | 0.40 | 0.50 |
|  | T2w+ADC (*C_Δr_*) | 0.42 | 0.46 | 0.63 |
|  | Imaging+Cl at baseline (*C_Δrbcl_*) | 0.83 | 0.78 | 0.77 |
|  | Imaging+ delta Cl (*C_ΔrΔcl_*) | 0.83 | 0.75 | 0.77 |

Specificity, PPV, and NPV computed at 70% sensitivity

**Supplemental-II: MRI Image standardization**

Image Standardization with example:


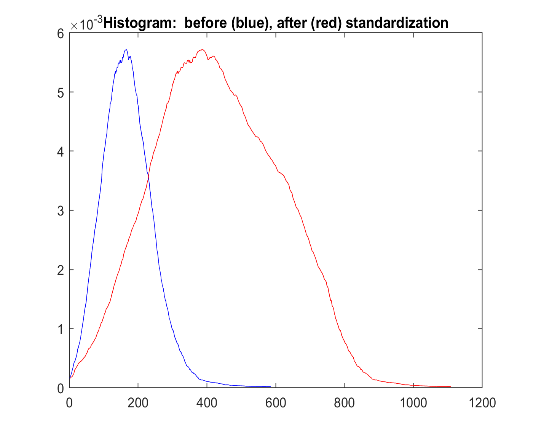

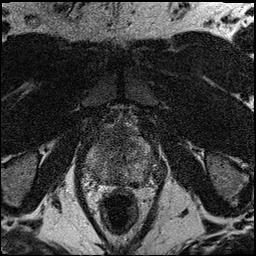

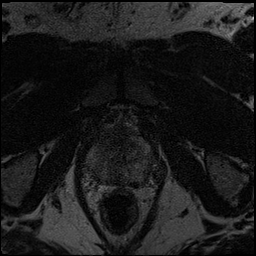


Smoothed histogram before and after standardization

Before standardization

After standardization

**Supplemental-III: Elastix parameters**

// This parameter file has kind of realistic values.

// In most other parameter files for testing, the number of samples and iterations is rather low, to allow fast testing.

// ********** Image Types

(FixedInternalImagePixelType "float")

(FixedImageDimension 3)

(MovingInternalImagePixelType "float")

(MovingImageDimension 3)

// ********** Components

(Registration "MultiResolutionRegistration")

(FixedImagePyramid "FixedRecursiveImagePyramid")

(MovingImagePyramid "MovingRecursiveImagePyramid")

(Interpolator "LinearInterpolator")

(ResampleInterpolator "FinalLinearInterpolator")

//(Metric "AdvancedNormalizedCorrelation")

(Metric "NormalizedMutualInformation")

(Optimizer "AdaptiveStochasticGradientDescent")

(Resampler "DefaultResampler")

(Transform "EulerTransform")

//(Transform "AffineTransform")

//(Scales 100000 10 10 10 100000 10 10 10 100000 1000 1000 1000 )

// ********** Pyramid

// Total number of resolutions

(NumberOfResolutions 3)

(ImagePyramidSchedule 4 4 4 2 2 2 1 1 1)

//(FixedImagePyramidSmoothingSchedule 8 8 8 4 4 4 2 2 2 1 1 1)

//(MovingImagePyramidSmoothingSchedule 8 8 8 4 4 4 2 2 2 1 1 1)

(ImagePyramidSmoothingSchedule 4 4 4 2 2 2 1 1)

// ********** Transform

(AutomaticScalesEstimation "true")

(AutomaticTransformInitialization "true")

(AutomaticTransformInitializationMethod "GeometricalCenter")

(HowToCombineTransforms "Compose")

// ********** Optimizer

// Maximum number of iterations in each resolution level:

(MaximumNumberOfIterations 500)

(AutomaticParameterEstimation "true")

(UseAdaptiveStepSizes "true")

// ********** Metric

(NumberOfHistogramBins 32)

(FixedKernelBSplineOrder 0)

(MovingKernelBSplineOrder 3)

// ********** Several

(WriteTransformParametersEachIteration "false")

(WriteTransformParametersEachResolution "true")

(WriteResultImageAfterEachResolution "false")

(WriteResultImage "false")

(ResultImageFormat "nii.gz")

(ShowExactMetricValue "false")

(ErodeMask "false")

(UseDirectionCosines "true")

(CheckNumberOfSamples "true")

// ********** ImageSampler

//Number of spatial samples used to compute the mutual information in each resolution level:

(ImageSampler "RandomCoordinate")

(NumberOfSpatialSamples 50000)

(NewSamplesEveryIteration "true")

(UseRandomSampleRegion "false")

(MaximumNumberOfSamplingAttempts 5)

// ********** Interpolator and Resampler

//Order of B-Spline interpolation used in each resolution level:

(BSplineInterpolationOrder 1)

//Order of B-Spline interpolation used for applying the final deformation:

(FinalBSplineInterpolationOrder 3)

//Default pixel value for pixels that come from outside the picture:

(DefaultPixelValue 0)

**Supplemental-IV: Radiomic feature map for the prostate region**

We extracted radiomic features from the prostate region and have shown the feature map.Radiomic features extracted from the entire prostate has significantly dilute the signal associated with pathological upgrade which is primarily driven by the lesion characteristics.


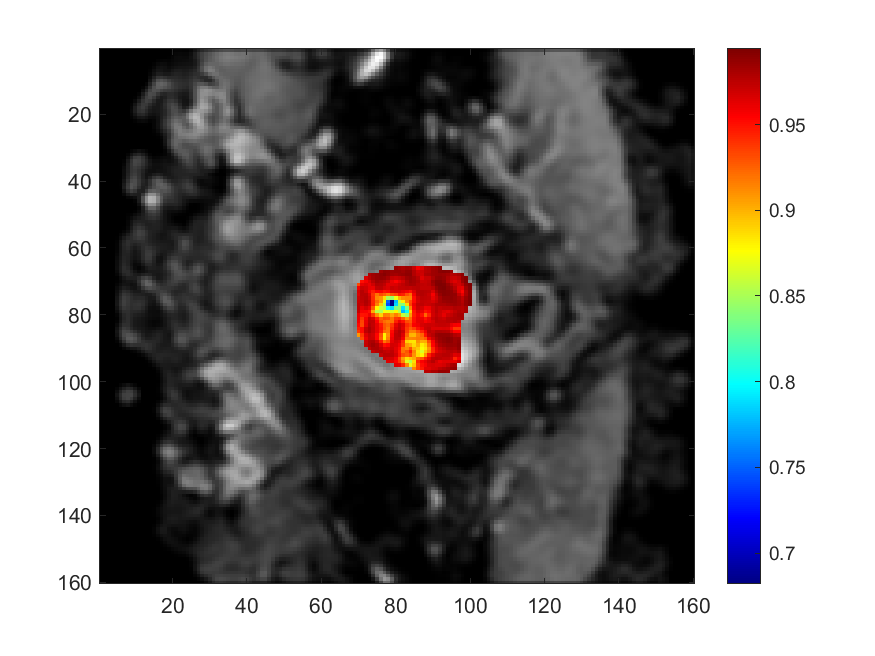

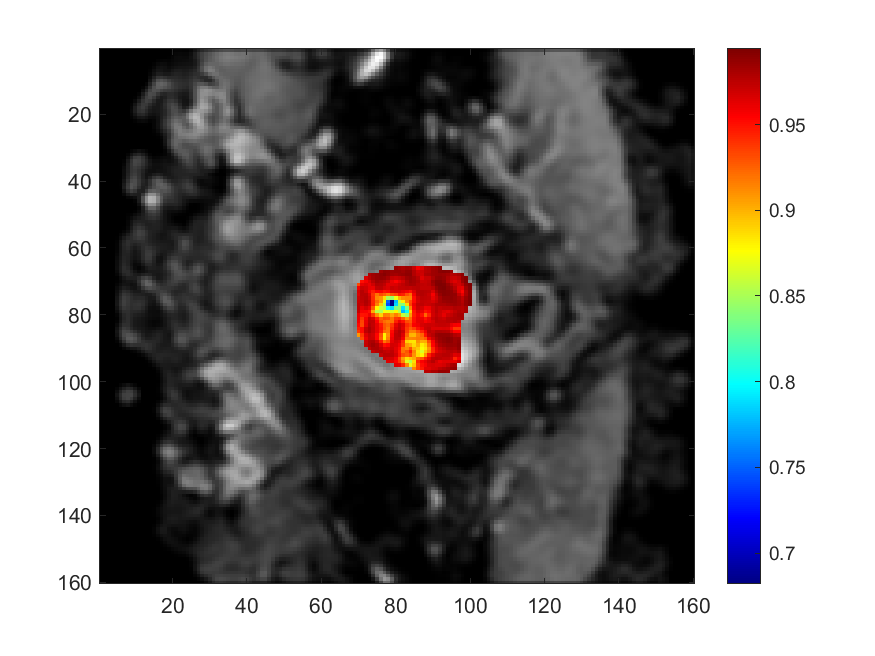

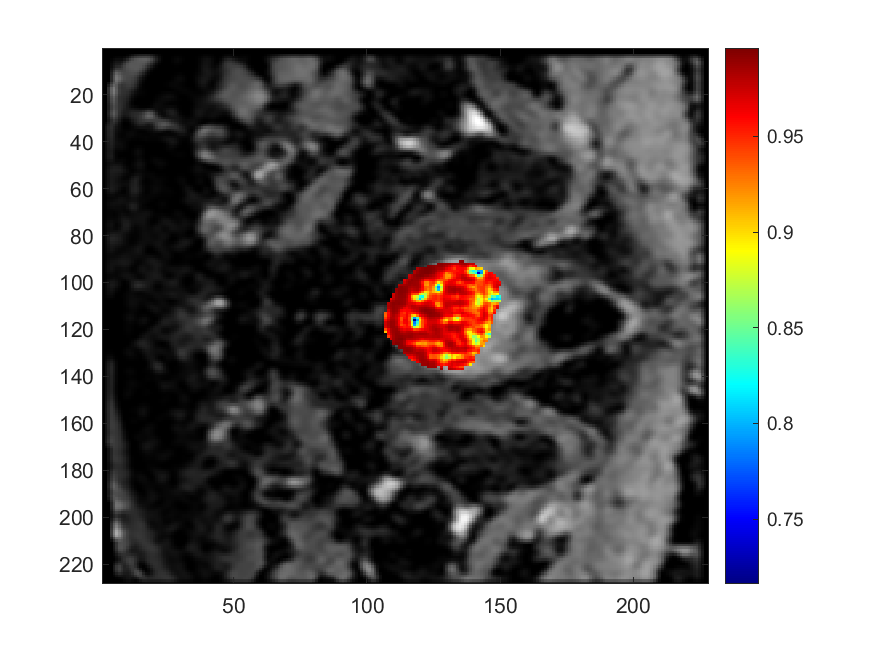

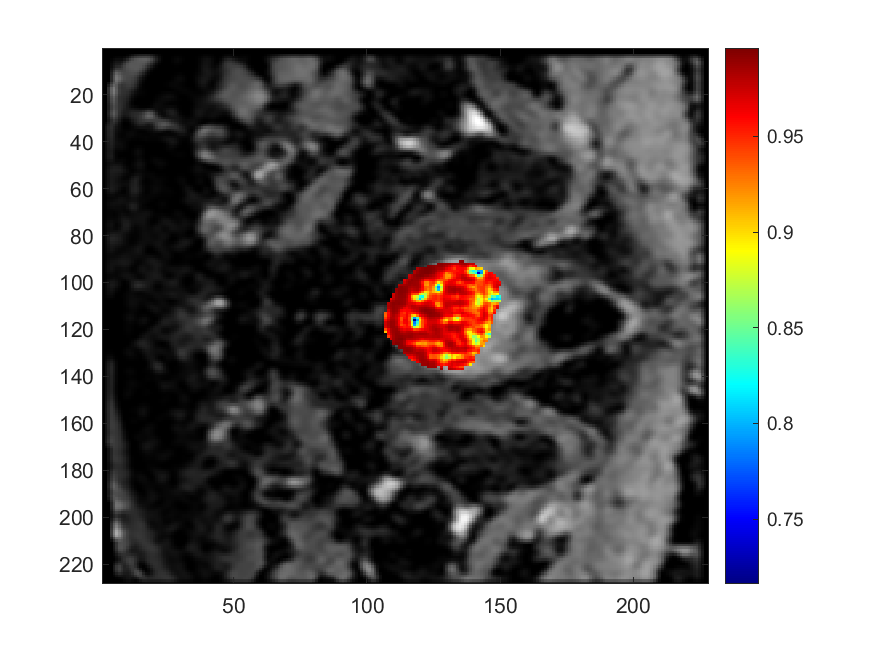

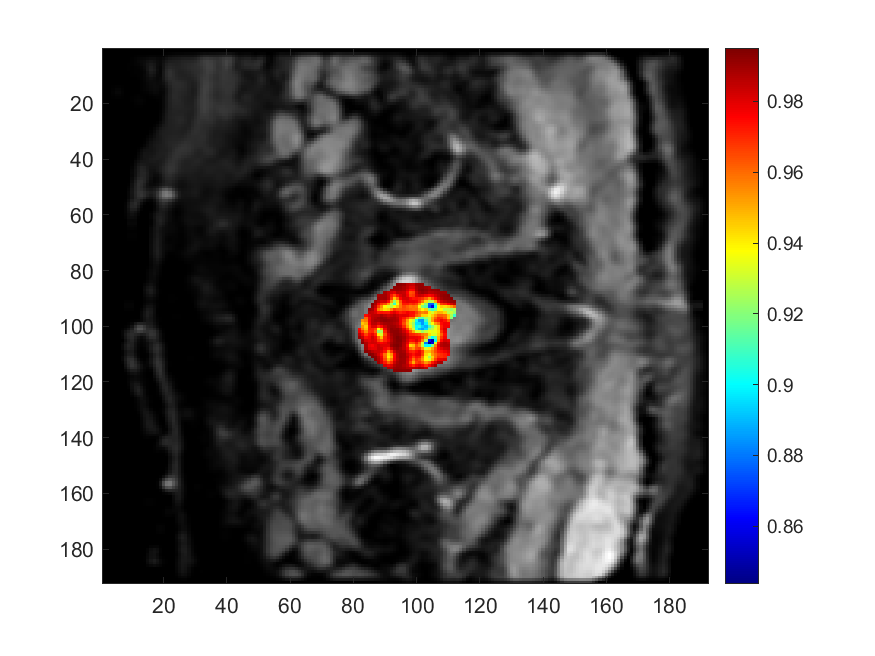

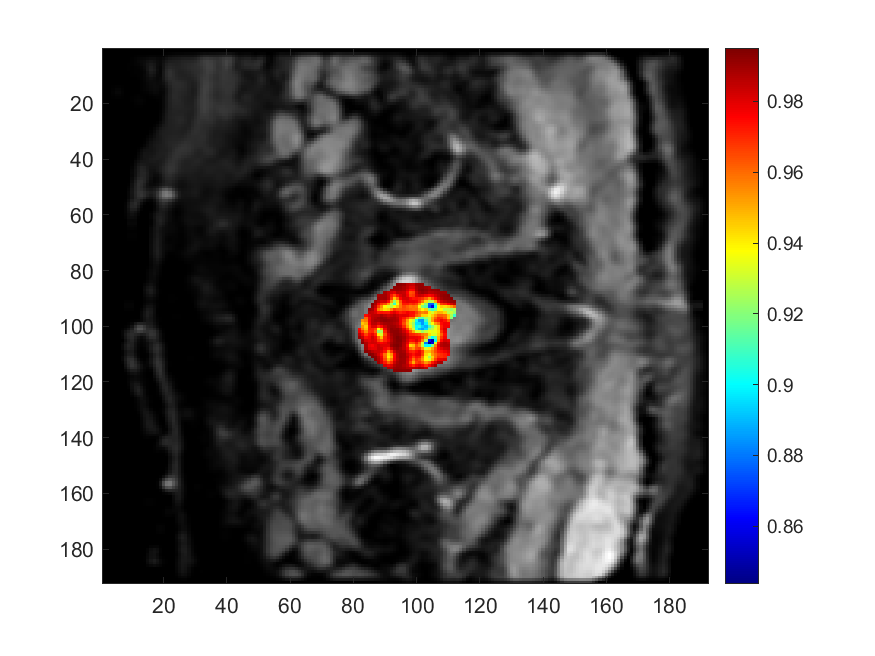

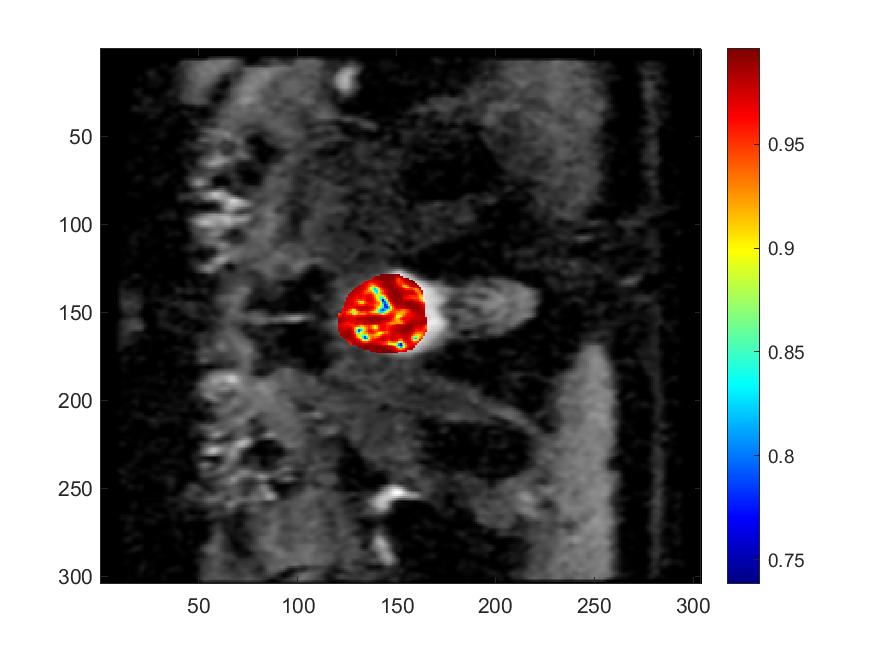

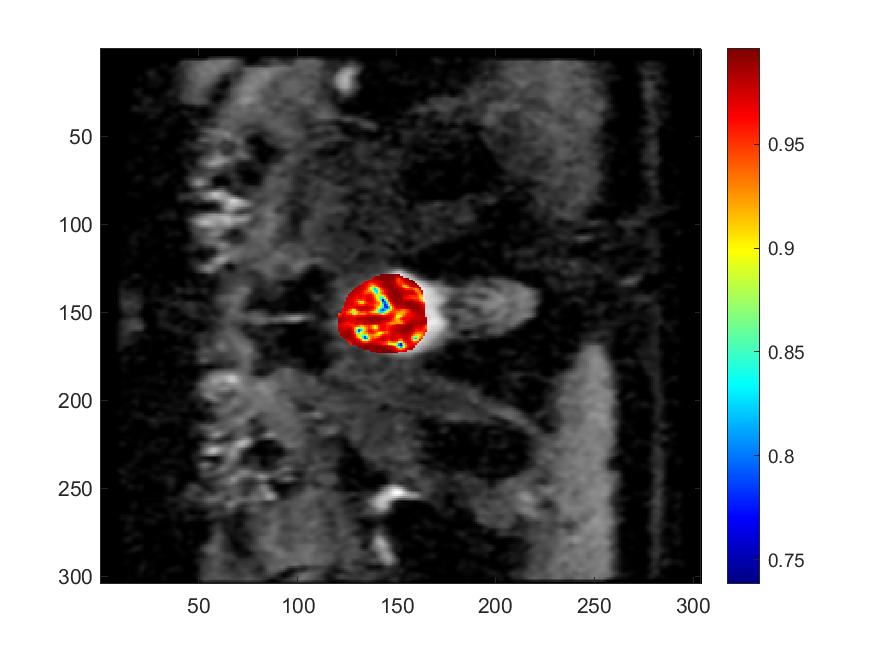

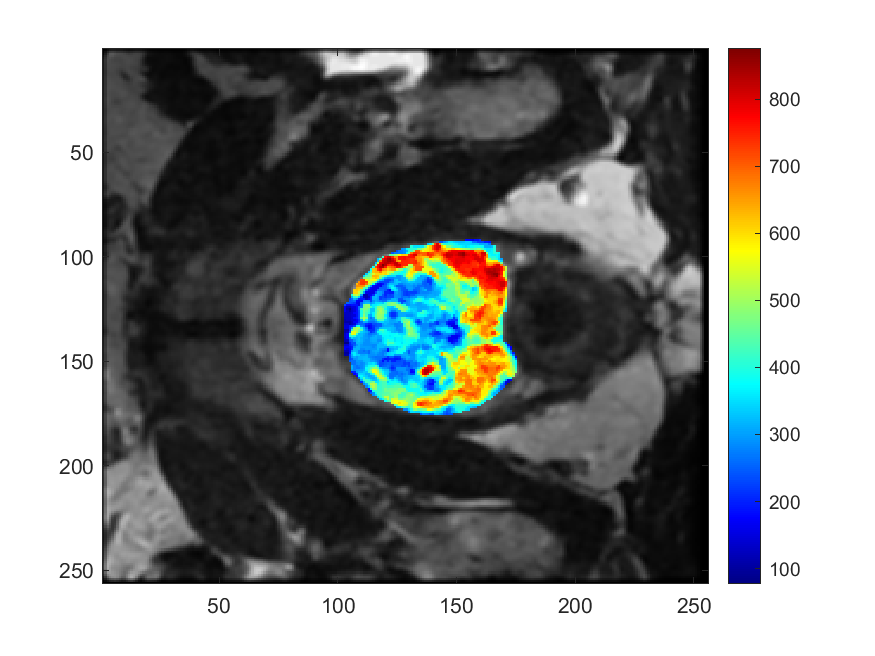

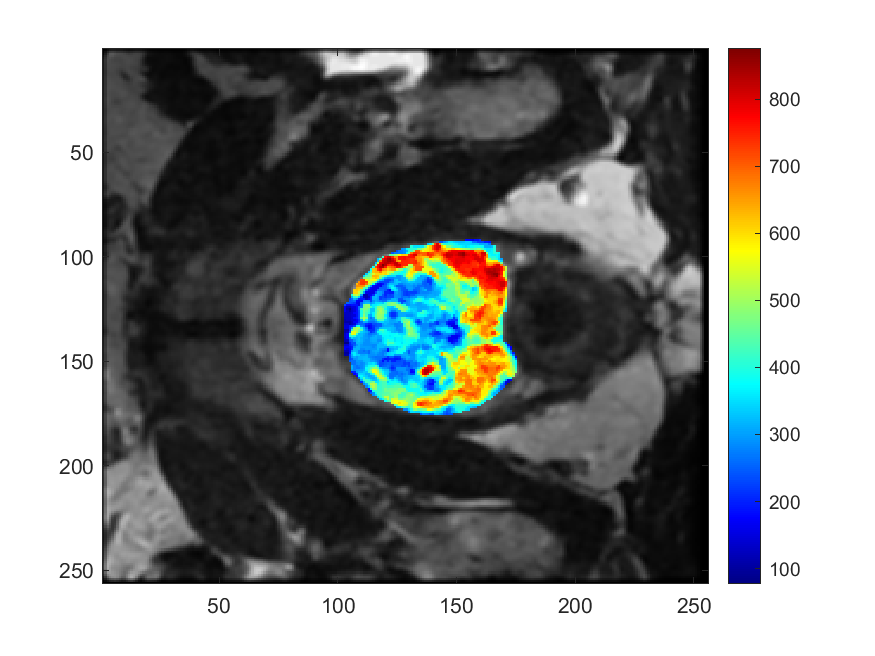

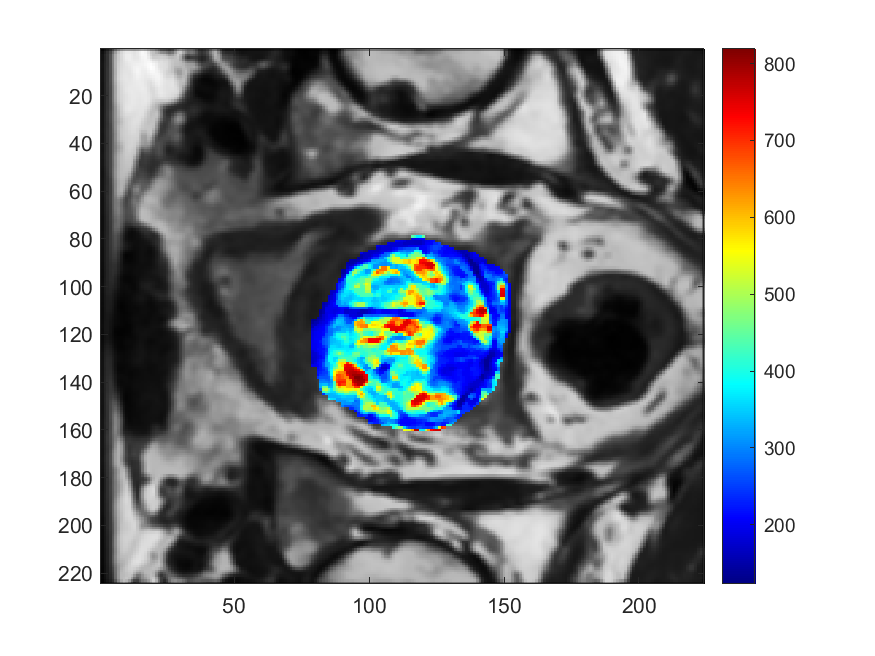

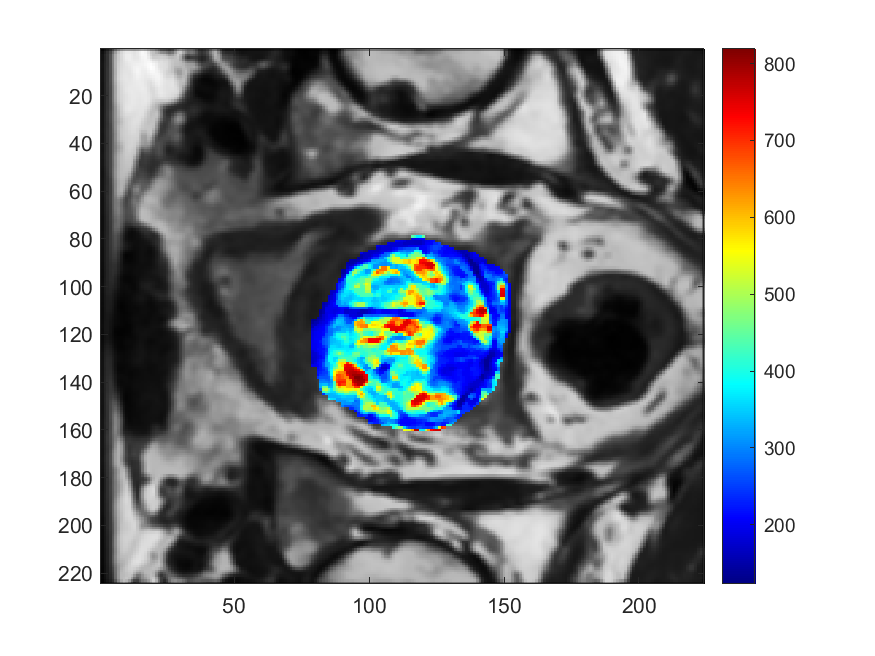

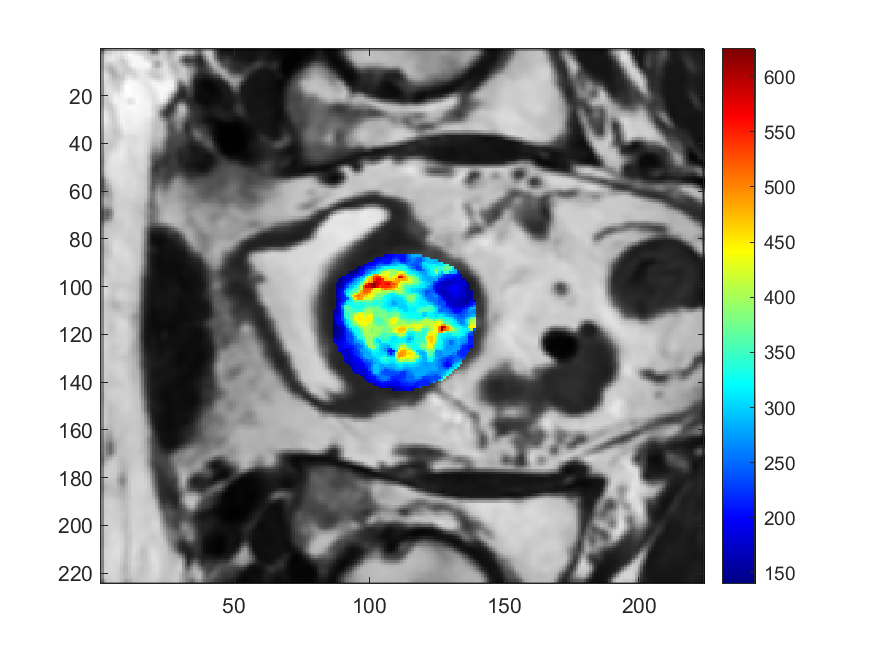

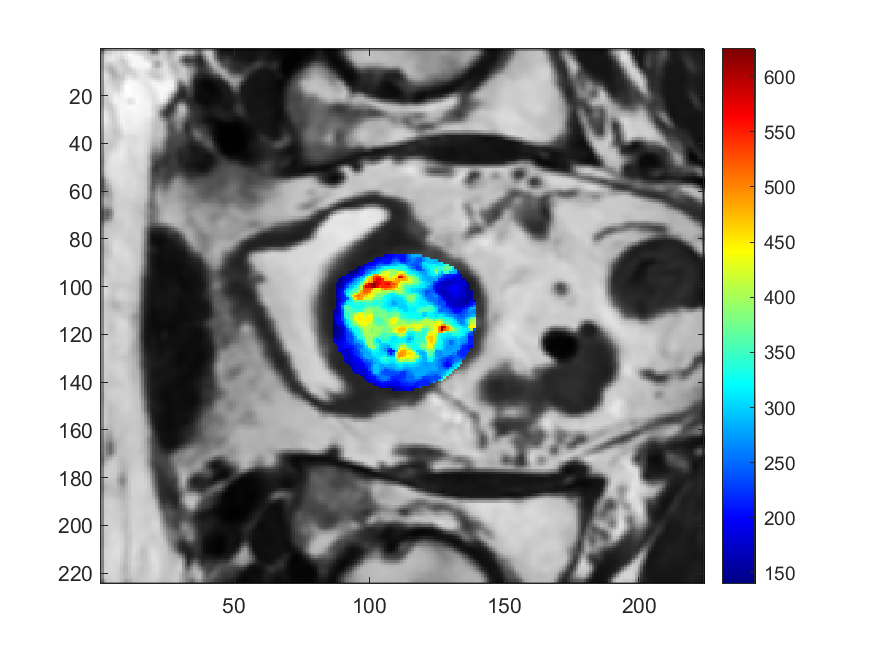

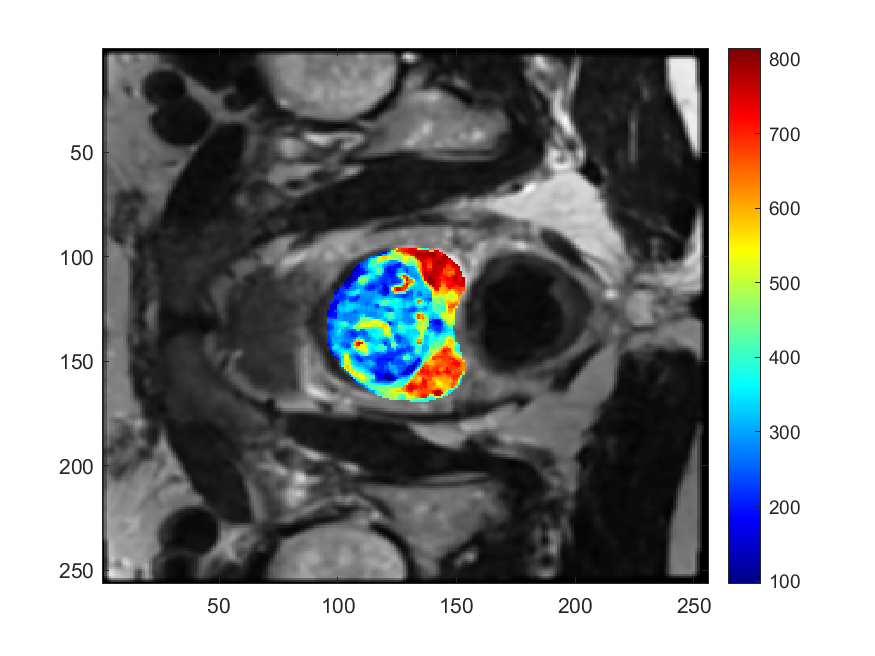

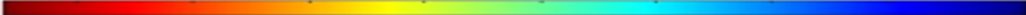


AS+

AS-

Prostate

Prostate

Tumor

Tumor

Follow-up MRI

Follow-up MRI

Baseline MRI

Baseline MRI

CoLIAGe (T2W)

Haralick  (ADC)


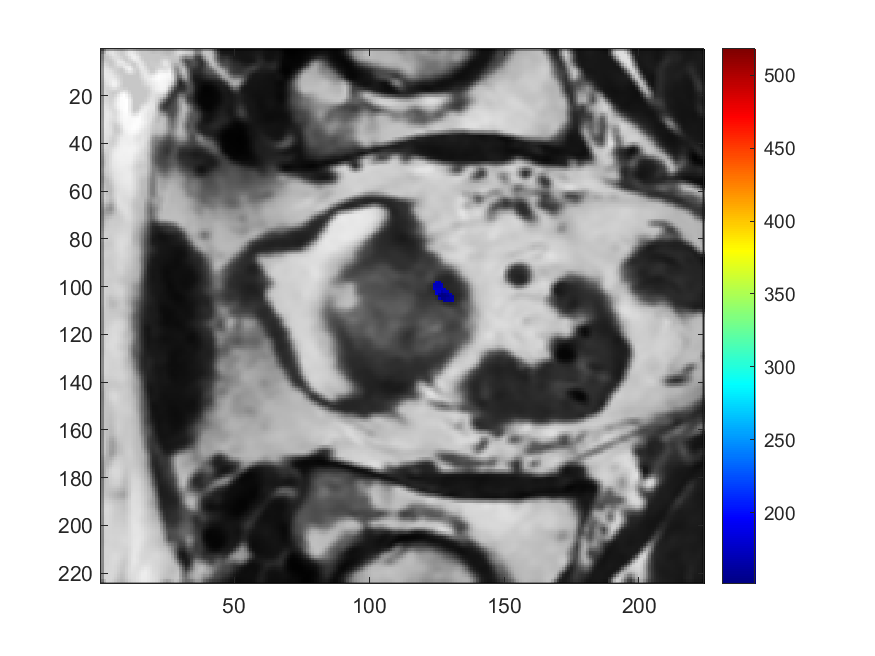

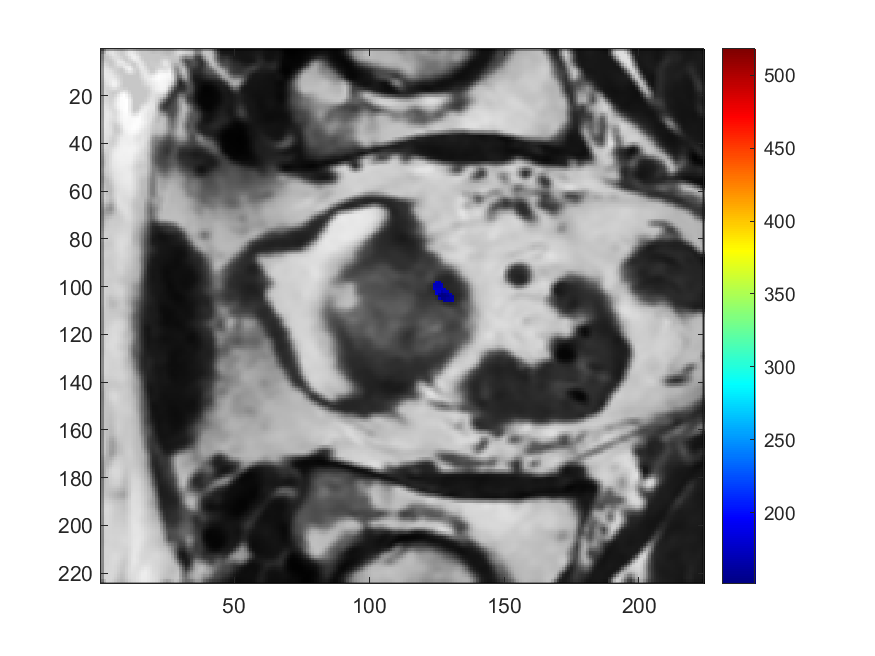

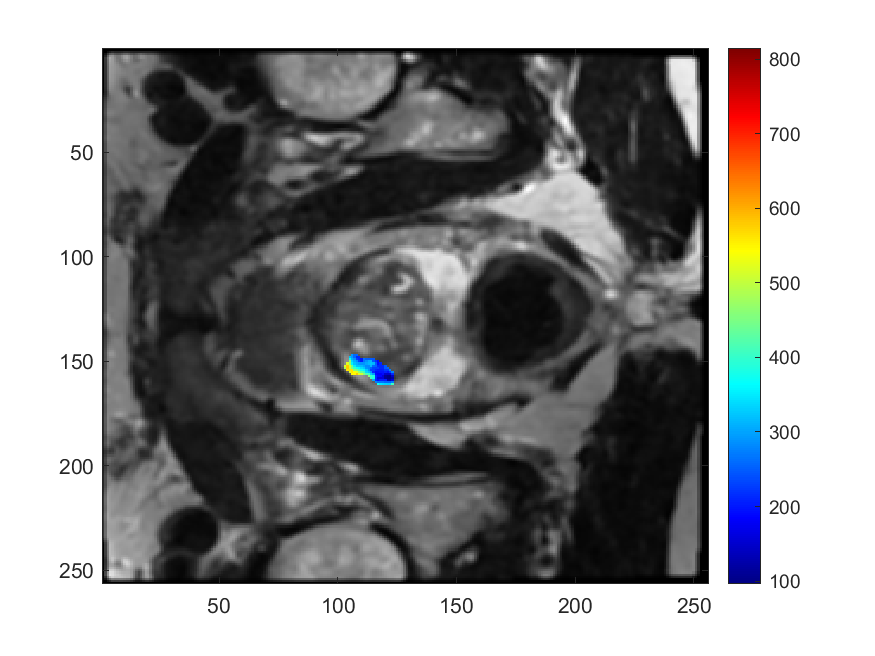

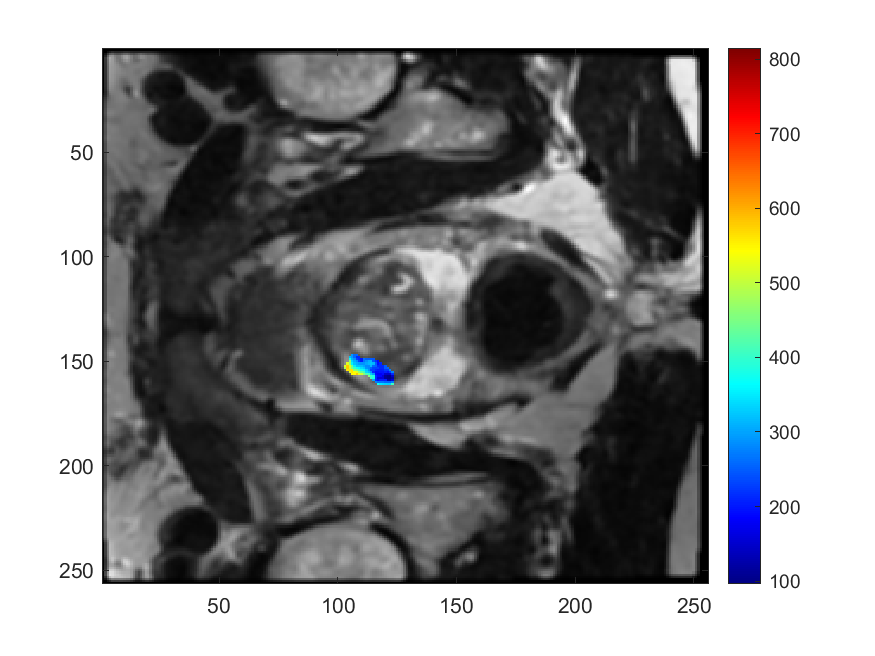

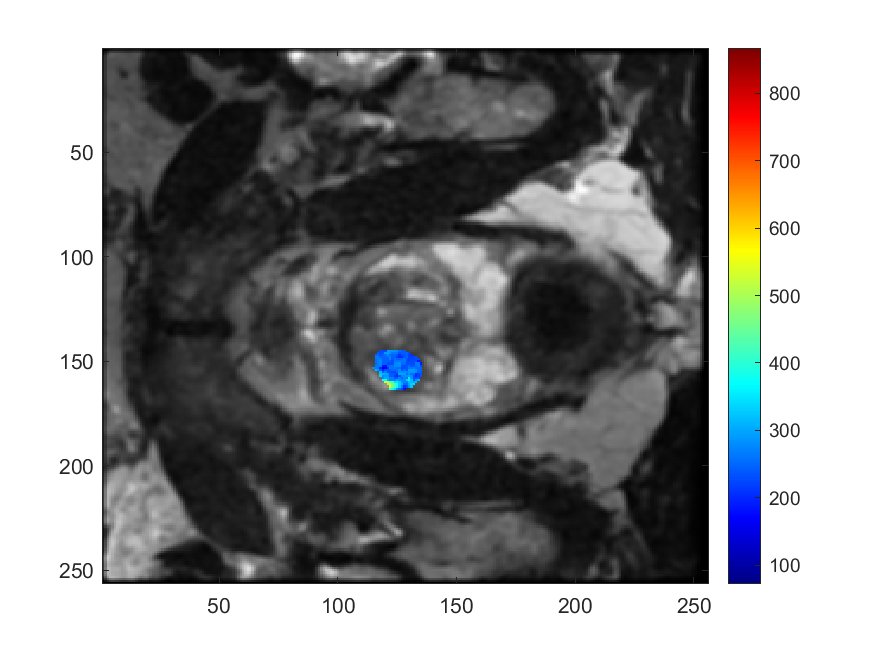

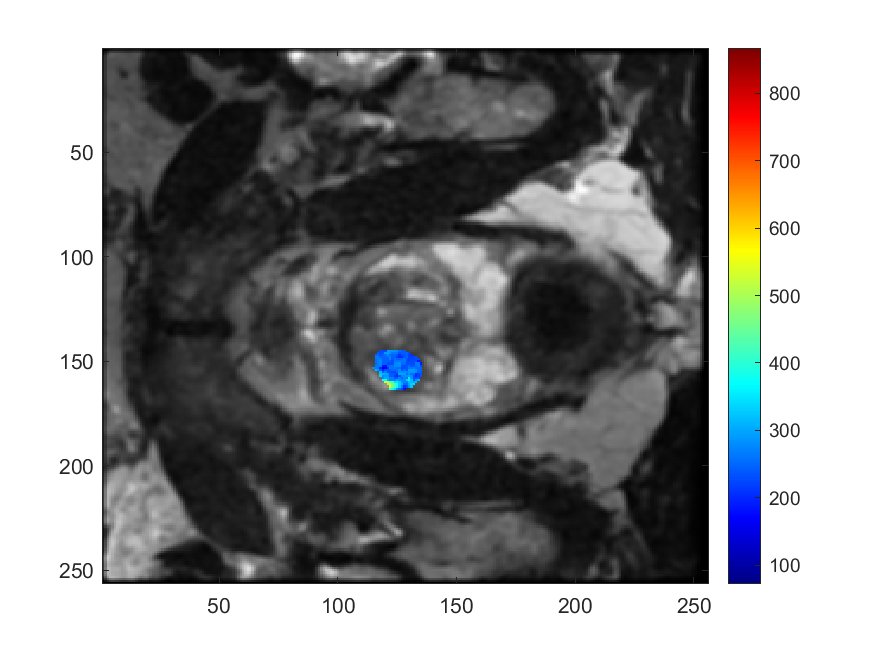

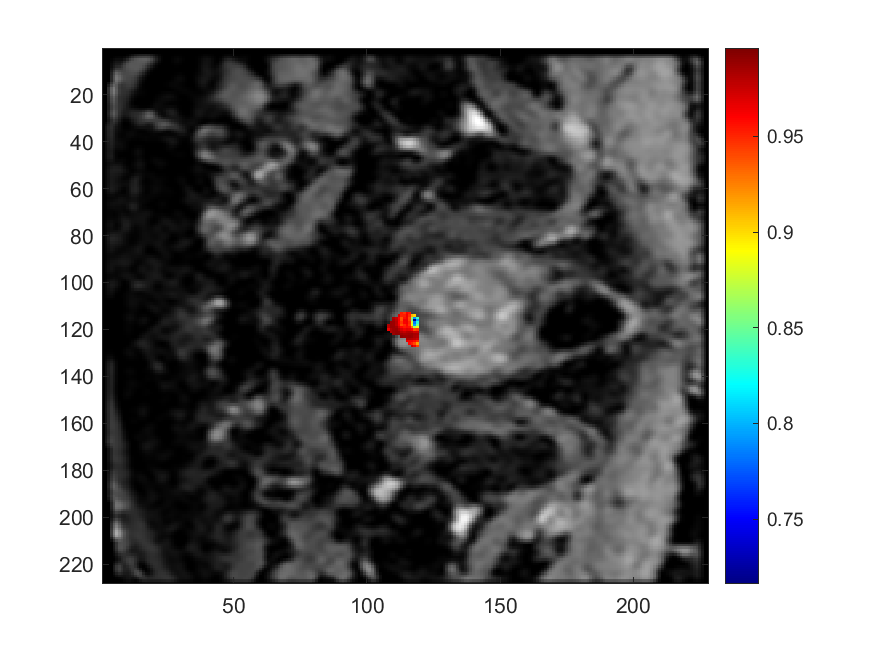

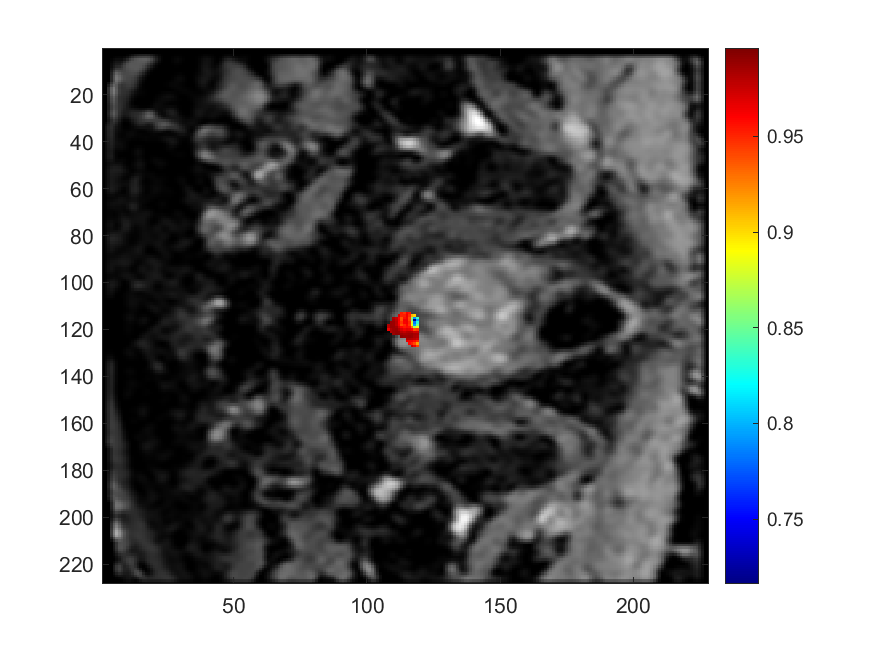

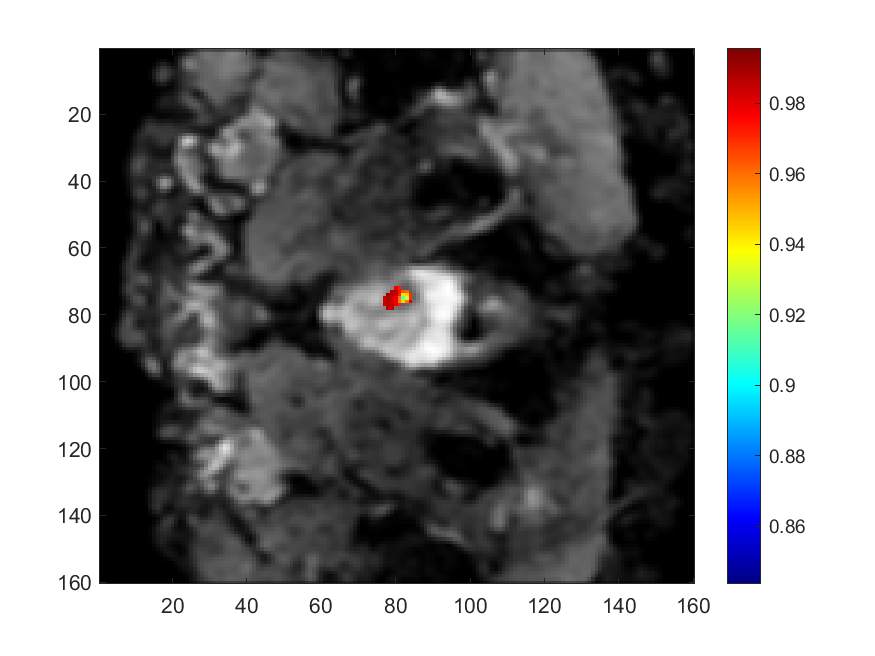

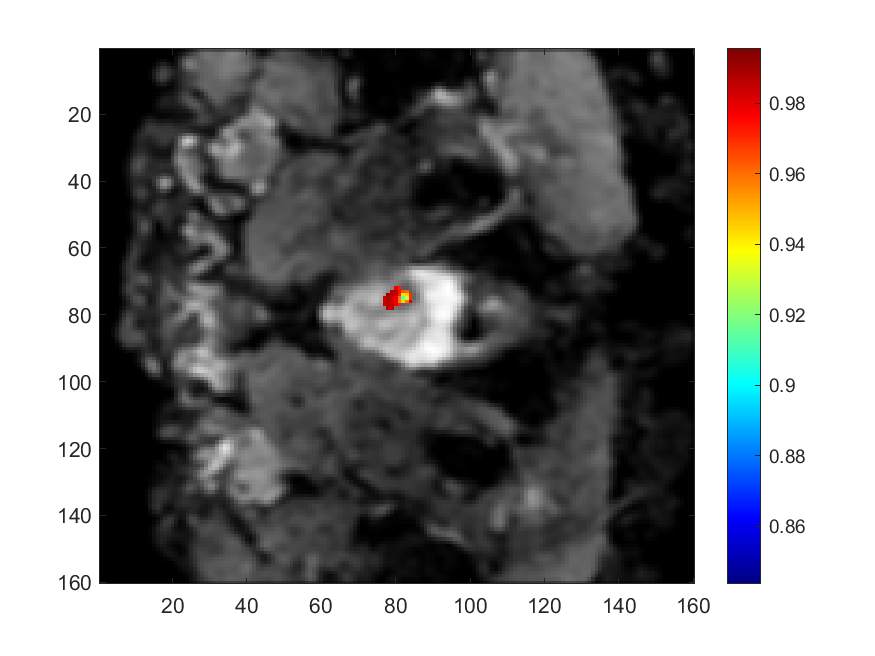

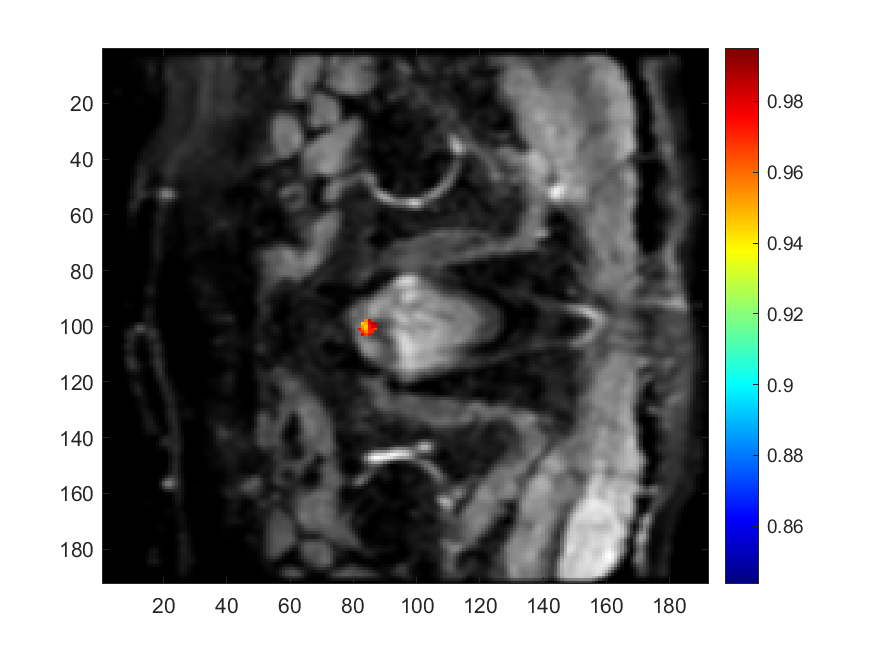

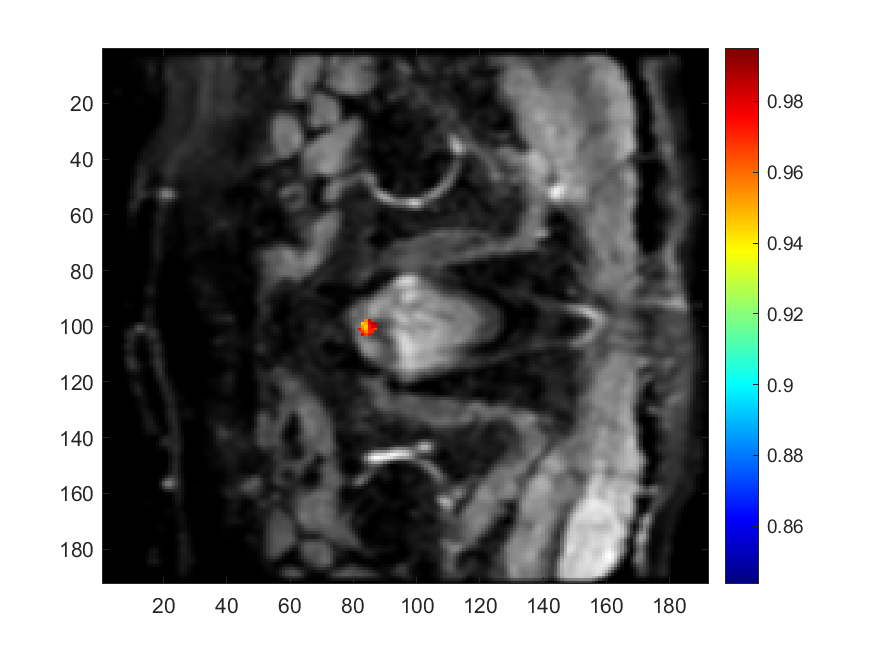

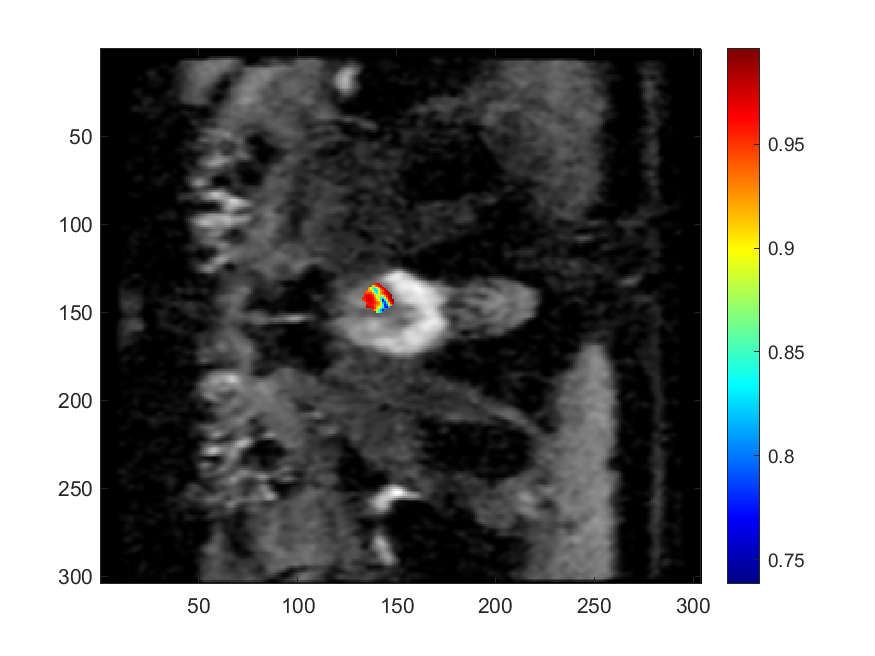

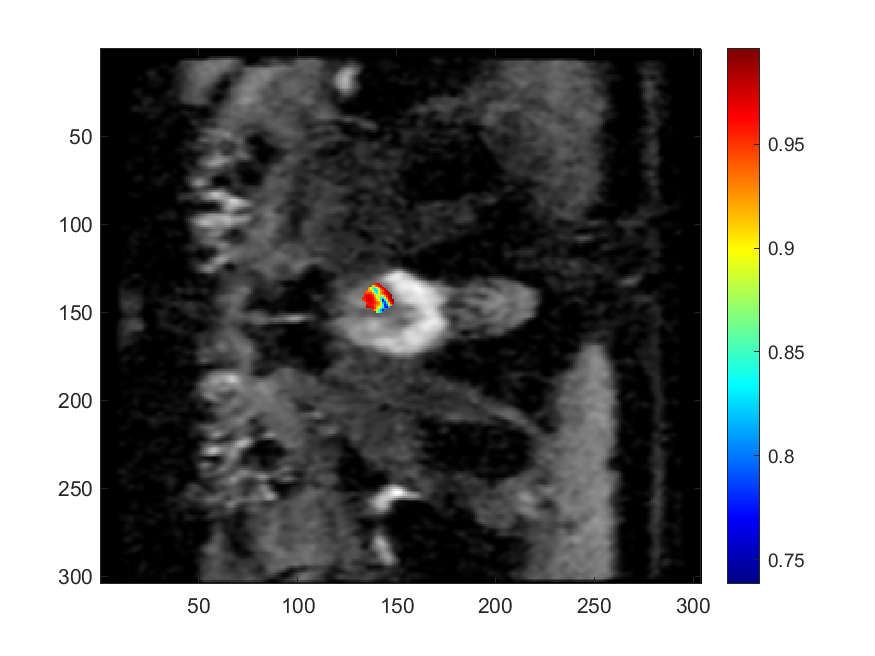

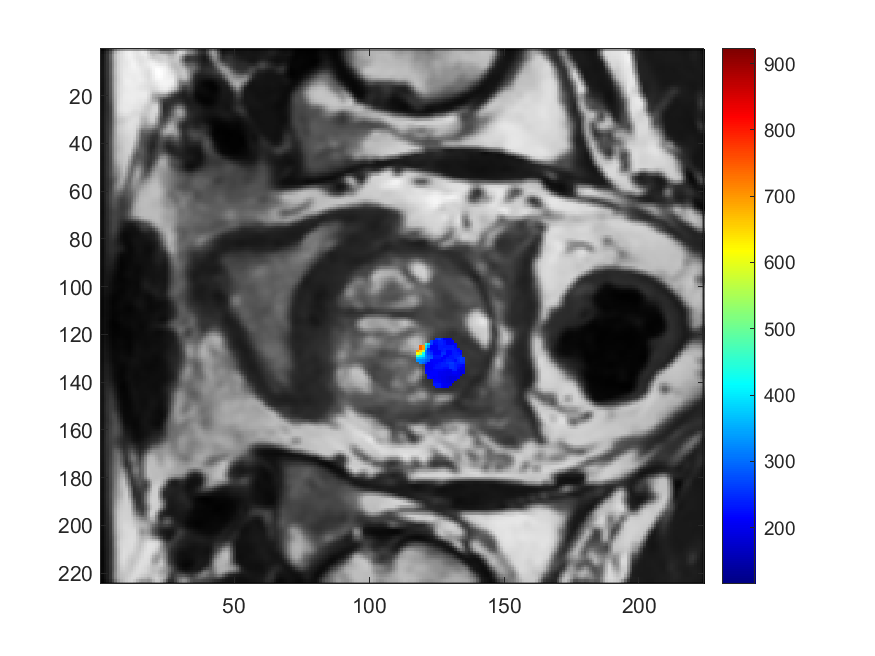

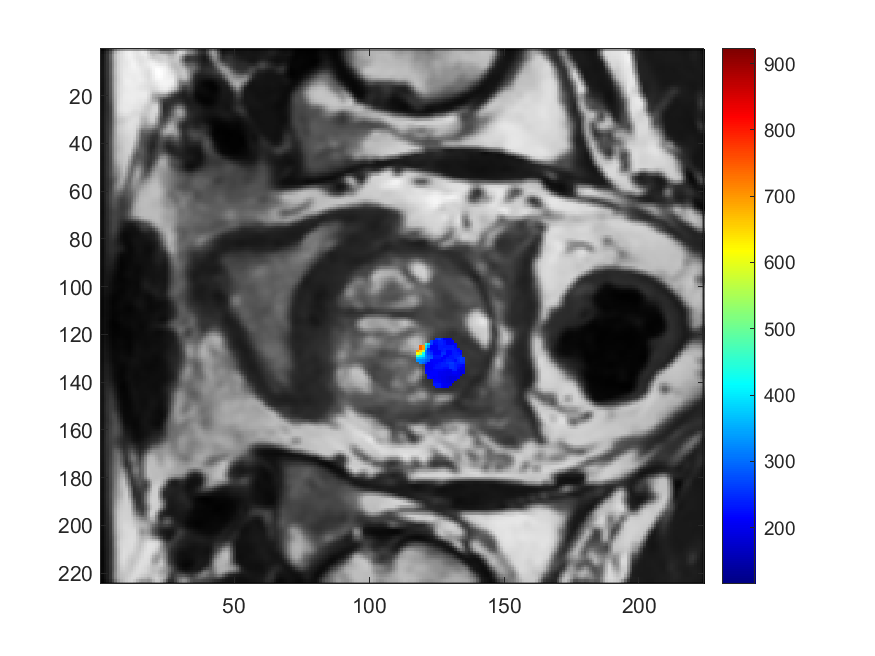


Figure: Radiomic feature maps of prostate region (row 1,3) and PCa (row 2,4) at baseline MRI and follow up MRI belonging to patients with pathologic progression (AS+) and those without (AS-). CoLIAGe features from T2W MRI (columns 1 and 2) and Haralick’s feature from ADC maps (columns 3,4) illustrated differential heterogeneity in appearance of prostate as well tumor region for AS+ and AS- patients between baseline and follow-up. The inset shows radiomic heat maps of the prostate region and PCa lesion in greater detail. Hotter colors (red) indicate higher heterogeneity associated with radiomic features and cooler colors indicate (blue) lower heterogeneity.
